# From Present Innovations to Future Potential: The Promising Journey of Lithium-Ion Batteries

**DOI:** 10.3390/mi16020194

**Published:** 2025-02-07

**Authors:** Pooya Parvizi, Milad Jalilian, Alireza Mohammadi Amidi, Mohammad Reza Zangeneh, Jordi-Roger Riba

**Affiliations:** 1 Department of Mechanical Engineering, University of Birmingham, Edgbaston, Birmingham B15 2TT, UK; pxp046@alumni.bham.ac.uk; 2 Department of Physics, Faculty of Science, Lorestan University, Khorramabad 4431668151, Iran; jalilianm70@gmail.com; 3 Pooya Power Knowledge Enterprise, Tehran 1466993771, Iran; alireza.moamidi@gmail.com (A.M.A.); m.zangeneh@alumni.sbu.ac.ir (M.R.Z.); 4 Department of Electrical Engineering, Faculty of Science, Razi University, Kermanshah 6714414971, Iran; 5 Department of Electrical Engineering, Universitat Politècnica de Catalunya, 08222 Terrassa, Spain

**Keywords:** lithium-ion batteries, anodes, cathodes, electrolytes, energy density, cycling stability, sustainability challenges, environmental impact, recycling practices

## Abstract

Lithium-ion batteries (LIBs) have become integral to modern technology, powering portable electronics, electric vehicles, and renewable energy storage systems. This document explores the complexities and advancements in LIB technology, highlighting the fundamental components such as anodes, cathodes, electrolytes, and separators. It delves into the critical interplay of these components in determining battery performance, including energy density, cycling stability, and safety. Moreover, the document addresses the significant sustainability challenges posed by the widespread adoption of LIBs, focusing on resource depletion and environmental impact. Various recycling practices, including hydrometallurgy, pyrometallurgy, and direct recycling, are evaluated for their efficiency in metal recovery and ecological footprint. The advancements in recycling technologies aim to mitigate the adverse effects of LIB waste, emphasizing the need for sustainable and scalable solutions. The research underscores the importance of ongoing innovation in electrode materials and recycling methodologies, reminding us of our responsibility and commitment to finding and implementing these solutions, as this continuous improvement is crucial to enhance the performance, safety, and sustainability of LIBs, ensuring their continued relevance in the evolving energy storage landscape.

## 1. Introduction

**Figure 1:**
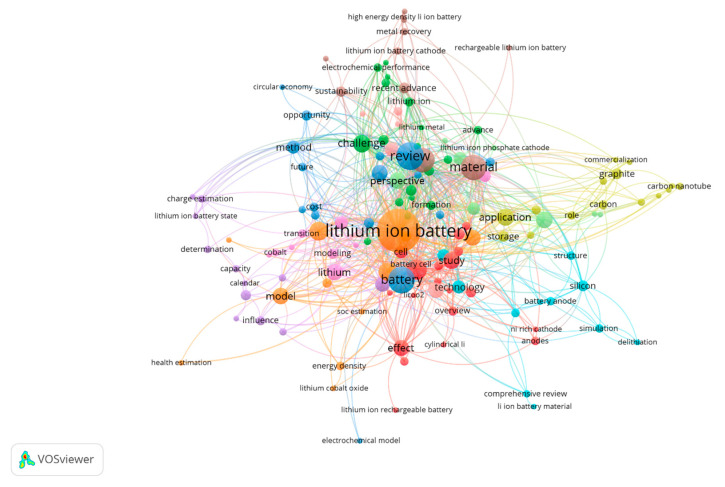
Clustered network visualization map of frequently used keywords by authors in titles, abstracts, and keywords.

Energy storage systems (ESSs) are pivotal to contemporary energy management, facilitating the effective utilization of renewable sources, bolstering grid stability, and fostering electric mobility. With the ever-increasing demand for energy, the necessity for reliable and efficient storage solutions becomes increasingly pronounced. A diverse array of energy storage technologies is available, such as pumped hydro storage, flywheels, supercapacitors, and chemical batteries. Each of these technologies plays a distinct role in enhancing energy resilience and sustainability, contributing significantly to the transition towards a more sustainable energy future [1,2,3,4,5]. Over the past few years, there have been substantial improvements in lithium-ion batteries (LIBs), leading to a surge in research efforts to address the significant gap in the battery market [6,7,8,9]. Since the 1990s, LIBs have maintained their dominance in the rechargeable battery market compared to other competitors [10,11]. In recent years, there has been an unprecedented surge in demand for high-performance rechargeable batteries, underscoring their indispensable role across diverse applications. This widespread recognition highlights their importance in advancing technologies and sustainability efforts globally [12,13,14]. The usage areas and market dominance of LIBs have expanded quickly and are consistently on the rise [15,16]. The industry has embraced and successfully commercialized numerous innovative materials [17]. The growth in battery research has been remarkable, mainly due to advancements such as miniature transistors. These innovations have significantly enhanced battery capabilities and increased their relevance across various industries [18]. LIBs are extensively employed as energy storage solutions in electronic devices, ESSs, and electric vehicles (EVs) [19,20,21,22,23]. Furthermore, global governments are increasingly recognizing the impact of greenhouse gases on climate change. They are backing advancements in green energy technologies such as solar, wind power, and energy-efficient EVs [4,24]. The growing popularity of wearable electronics heavily influences the future trajectory of LIBs. Present-day researchers have introduced significant factors related to battery weight, size, extended lifespan, safety, and reduced costs, which are now essential considerations in battery manufacturing [24,25,26]. Three decades ago, Sony Co. achieved a milestone by commercializing the world’s inaugural LIB, heralding a transformative era in portable electronics [20,27,28]. This breakthrough triggered an unprecedented field of study, amplified by global governmental initiatives addressing climate change through green energy technologies and EVs, where ESSs play a pivotal role. Over the past seven years alone, publications on batteries have surged by 260%, reflecting a rapid expansion in research endeavors, far outstripping the growth rate of general literature [29,30]. Figure 1 illustrates the most prominent and up-to-date topics within the field, categorized by their frequency of occurrence. Table 1, derived from the scientific literature, presents various key performance indicators (KPIs) that describe the characteristics of LIBs.

This review provides crucial insights into the future of battery technology, focusing on the technical challenges in developing LIBs and evaluating global market trends. It emphasizes the increasing interest in alternative energy storage solutions, such as lithium-air and lithium-sulfur batteries (LSBs), alongside the ongoing importance of LIBs. Despite their advantages in energy density and fast charging, LIBs encounter significant issues, including safety concerns, manufacturing challenges, and environmental impacts. While progress has been made in reducing costs and enhancing energy density, concerns persist regarding sustainable supply chains, particularly with the expanding EV market. Safety issues like thermal runaway and environmental impacts from production and disposal further complicate matters. Recycling technologies hold promise but need help optimizing recovery rates and managing pollutants. The study traces the transformative journey of LIB technology from its inception during the oil crisis to its pivotal role across diverse applications today. Advances in material science and electrode engineering, coupled with rising demand for high-performance rechargeable batteries, underscore the importance of continuous research and development in shaping the future of energy storage technologies. Overall, this comprehensive review offers a detailed perspective on LIBs, addressing structural aspects, recent advancements, adoption challenges, and future research trajectories, providing valuable insights for stakeholders in advancing energy storage solutions. The findings offer useful insights for researchers, policymakers, and industry stakeholders engaged in developing and deploying advanced energy storage solutions.

### 1.1. Market Overview

**Figure 2:**
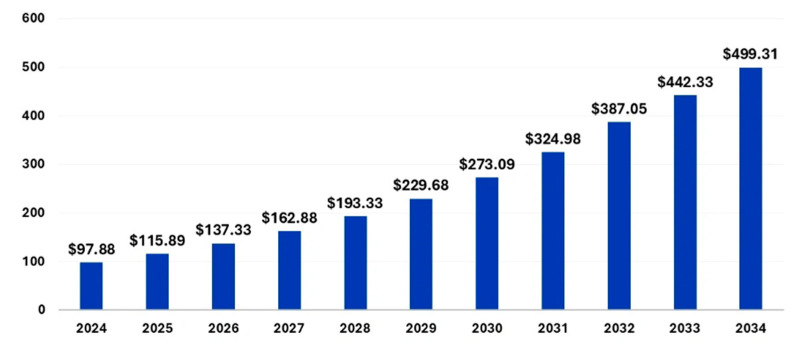
Forecasted market size for LIB during 2024 to 2034 (in USD billion) [32].

The global LIB market is experiencing robust growth, with an estimated size of USD 97.88 billion in 2024, projected to reach USD 499.31 billion by 2034, reflecting a compound annual growth rate (CAGR) of 17.69% from 2025 to 2034 (see in Figure 2). This expansion is driven primarily by the increasing demand for EVs, the rising adoption of consumer electronics such as smartphones, and growing disposable incomes. The Asia–Pacific region dominates the market, holding a 53% market share in 2024, with China as a major production hub. North America is expected to be the fastest-growing market during the forecast period. The market is influenced by several key factors. The burgeoning automobile sector, particularly the EV segment, is a major growth driver due to the high energy density and efficiency of LIBs. Several leading manufacturers of EV batteries include Panasonic, Robert Bosch, SAMSUNG SDI, Beijing Pride New Energy Technology, BYD, Daimler, Mitsubishi, and Tianjin Lishen Battery. Other EV battery manufacturers include TESLA, Nissan, and Toyota. Government policies and incentives promoting EV adoption, coupled with a dramatic decrease in battery prices, are propelling this growth. Battery prices have fallen significantly, reaching approximately USD 139 per kilowatt hour in 2023, an 82.17% reduction from 2013 [32]. The use of these batteries in consumer electronics and the growing demand for renewable energy storage further boost the market. Technological advancements, mass production, and increased competition are leading to price declines, facilitating widespread adoption. However, the scarcity and concentrated production of key materials like lithium and cobalt pose potential restraints. Additionally, research into alternative battery chemistries using other metals is ongoing. The market is also seeing increasing collaborations between major players and government agencies to expand its reach and adoption [33]. In terms of products, lithium iron phosphate (LFP) batteries dominated in 2024, driven by demand for portable devices requiring durable, safe, and long-lasting batteries. Lithium nickel manganese cobalt (NMC) batteries are the fastest-growing segment, favored for their performance in applications like EVs. The electric and hybrid vehicle sector is the largest application segment, and ESS are also projected to grow significantly. The push towards environmental sustainability and renewable energy sources is driving demand for both EV batteries and energy storage solutions, with governments worldwide implementing policies and incentives to promote this shift [32]. Artificial intelligence (AI) is playing an increasing role in the development and manufacturing of LIBs. AI can help identify new compounds to enhance efficiency and optimize performance, as well as improving manufacturing processes. AI and machine learning are also used in smart battery systems that provide real-time monitoring and predictive maintenance [34]. The market also has a strong opportunity in the growing demand for environmental sustainability, with governments pushing for the adoption of LIBs to reduce reliance on internal combustion engines. The recyclability of these batteries further adds to their appeal [32].

### 1.2. Historical Context

**Figure 3:**
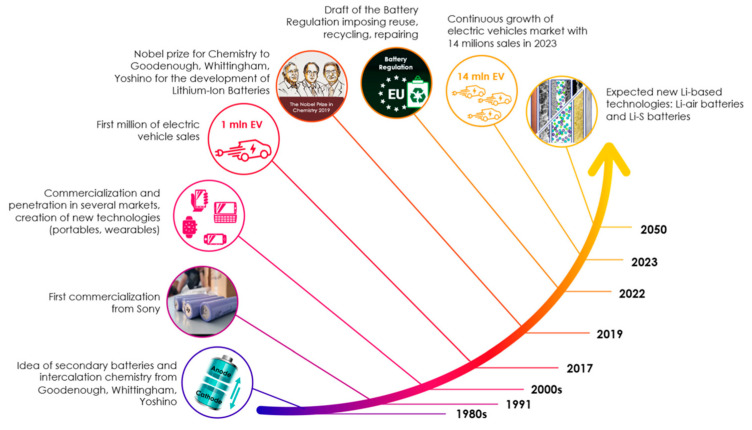
Outline of the historical development and prospects of LIBs. The successful commercialization of LIBs has enabled their extensive use in diverse applications, ranging from portable electronics to EVs and ESSs [36].

The development of the LIB, a ubiquitous power source in modern electronics and EVs, is a story of continuous innovation spanning several decades. The initial concept emerged in the 1970s, with scientist M. Stanley Whittingham exploring the use of lithium metal and titanium disulfide in battery prototypes. However, these early designs were hampered by safety issues and the high cost of materials. The 1980s proved to be a pivotal decade, marked by significant breakthroughs. A key discovery was the ability to intercalate lithium ions into graphite, which allowed for the development of safer and more stable anodes. This breakthrough, along with the exploration of alternative cathode materials such as manganese spinel, paved the way for the creation of more practical batteries. A landmark achievement came in 1985 when Akira Yoshino created a prototype using lithium cobalt oxide (LiCoO_2_), a material that significantly improved battery performance and safety. Notably, in 1991, Sony released the first commercially available LIB, which utilized layered oxide chemistry, further promoting the technology’s adoption. The use of nanomaterials, starting in the late 2000s, further boosted battery performance by increasing surface area and improving capacity and cycle life. Subsequent improvements focused on enhancing conductivity and performance through nanomaterials and doping techniques, as demonstrated by Yet-Ming Chiang’s work in 2002 and 2004. Since then, research has continued to refine lithium-ion technology. The ongoing development of lithium-ion technology promises to play a crucial role in powering a more sustainable future [35]. As illustrated in Figure 3, the history of LIBs has witnessed several significant milestones.

### 1.3. Fundamentals of LIBs

A LIB is created by linking essential lithium-ion cells together in parallel (to increase current), in series (to increase voltage), or in combined arrangements. Multiple battery cells can be incorporated into a module, and several modules can be combined into a battery pack [37]. A battery comprises an anode, a cathode, a separator, an electrolyte, and two current collectors (positive and negative). The fundamentals of LIBs encompass the intricate interplay between electrode materials, electrolytes, and the electrochemical processes governing charge and discharge cycles [38]. At the heart of LIBs are the anode and cathode, where lithium ions shuttle between during the charging and discharging phases [39,40]. The choice of electrode materials profoundly impacts the battery’s performance characteristics, such as energy density, cycling stability, and rate capability [12,41,42]. The electrolyte in a LIB, which usually consists of a lithium salt dissolved in an organic solvent, plays a crucial role in enabling the movement of lithium ions between the electrodes during charging and discharging cycles. Concurrently, the separator is designed to maintain physical separation between the electrodes to prevent short circuits while still allowing ionic conductivity. To ensure that LIBs deliver optimal performance and maintain safety standards, it is essential to thoroughly understand and carefully engineer various elements, such as ion transport mechanisms, the interactions at the electrode–electrolyte interfaces, and the appropriate voltage range within which the battery operates [43]. Researchers [12,18,27,29] continually explored novel electrode materials, electrolyte formulations, and battery architectures to enhance energy density, cycle life, and safety while mitigating environmental impacts. Advancements in LIB technology underpinned the widespread adoption of portable electronics. They drove the electrification of transportation and integration of renewable energy sources into the grid, highlighting the importance of ongoing research and development in this field. Advantages of LIBs include their high energy density, fast charging capability, versatility for various applications, and long cycle life [18,44,45]. LIBs are recognized for their versatility in design and technological advancement compared to other rechargeable batteries [46]. They excel in gravimetric and volumetric energy density, enabling them to be customized into a wide range of shapes and sizes to efficiently accommodate the available space in the devices used to power [47]. The LIB is a strong contender for future energy storage due to its impressive energy density, specific energy, and rechargeability [48]. LIBs have become the leading choice for energy storage in contemporary portable consumer electronics, including laptops, smartphones, and tablets. Their appeal extends beyond consumer electronics, as they are also considered the leading battery technology for fully electric and hybrid EVs and a viable option for stationary storage systems. Additionally, they are a strong candidate for stationary storage solutions. As a result, LIBs are extensively utilized in numerous personal and industrial applications, underscoring their flexibility and efficiency in addressing a wide range of energy storage needs [49,50,51,52]. However, they also pose safety concerns such as risks of overheating and thermal runaway, come with high manufacturing costs impacting affordability, face issues regarding resource depletion, raise sustainability questions, and contribute to environmental impact throughout their lifecycle. Further explanations about the principles and operation of LIBs are discussed in later chapters.

## 2. Basic Principles of LIB

### 2.1. Basic Principles of LIB Operation, Including Charge–Discharge Mechanisms and Ion Transport Electrode Materials and Their Role in Determining Battery Performance

The operational principles of LIBs include the processes of charge and discharge and the transport of ions within the battery [53,54,55,56,57,58]. During the charging phase, lithium ions migrate from the cathode (positive electrode) to the anode (negative electrode) through the electrolyte, where they are stored [59,60]. This migration is accompanied by the cathode material’s oxidation and the anode material’s reduction. In contrast, during the discharging phase, lithium ions move back to the cathode, releasing energy that can be used to power connected devices or systems. The electrolyte facilitates this bidirectional movement of lithium ions, enabling ion flow while simultaneously inhibiting electron flow, ensuring the battery’s electrical balance [42,61]. Table 2 illustrates the movement of lithium ions and electrons throughout the battery’s operation during both the charging and discharging processes. Emphasizing the significance of LIB performance and design optimization, Sepasi, Roose, and Matsuura [62] stated that ion transport within the battery was crucial for its performance. The conductivity of the electrolyte plays a pivotal role in determining the efficiency of lithium-ion transport between the electrodes during the charging and discharging processes. Additionally, Chen et al. [63] said that the choice of electrode materials impacted ion transport, as materials with higher diffusivity and lower resistance facilitated faster charging and discharging rates. Understanding these principles was essential for optimizing LIB design and performance, particularly in terms of energy density, cycle life, and safety. By highlighting the broader implications of LIB technology beyond operational principles, emphasizing the importance of addressing sustainability and environmental concerns for the continued advancement and adoption of LIBs, there were significant concerns regarding the sustainability and ecological impact of LIBs [64]. The demand for lithium and other essential raw materials, such as cobalt and nickel, has raised significant concerns regarding the sustainability of supply and the management of resources over the long term. These materials are crucial for producing LIBs, which power many modern technologies, from smartphones to EVs [65]. Furthermore, recycling LIBs has become a focus of research to alleviate material demand and reduce waste. Alternative battery chemistries, such as sodium-ion batteries, were also explored as potential solutions to address supply chain issues and environmental concerns associated with LIBs [66]. Addressing these challenges is paramount to ensuring LIB technology’s ongoing development and widespread use in diverse applications such as consumer electronics, EVs, and grid ESSs. The perspectives of Fleckenstein, Bohlen, and Bäker [67], along with Stokes et al. [68], provided valuable insights into the technical aspects and challenges of LIBs, emphasizing the importance of clear communication and understanding for sustainability. Wang et al. [69] highlighted the need for additional context to improve reader comprehension, especially for those less familiar with battery terminology. By integrating these viewpoints, a more comprehensive understanding of the significance of LIBs in contemporary technology can be achieved, addressing both technical intricacies and accessibility concerns [70]. This discussion on LIB technology and its implications is further supported by sources [71,72].

### 2.2. Electrode Materials and Their Role in Determining Battery Performance

**Figure 4:**
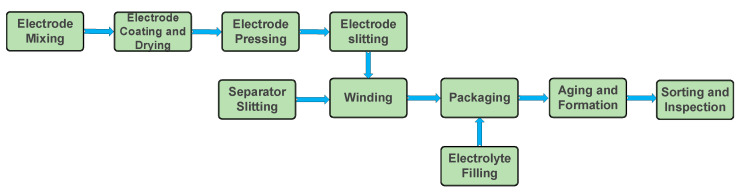
Flowchart depicting the process for fabricating electrodes [82] (reproduced with permission from Elsevier).

The growing demand for advanced materials and energy consumption has led to the pursuit of high-performance energy systems such as lithium-metal batteries, supercapacitors, and fuel cells [73]. LIBs in particular have become the preferred power source for portable devices, EVs, and a range of applications from grid storage to electric aircraft [74,75]. For a long time, the standard electrodes used in batteries have predominantly been made from inorganic materials [76]. Intense research efforts have resulted in the development of diverse electrode materials and a deep understanding of the structure–property relationships that influence their performance. Extensive efforts have focused on creating rechargeable batteries with new electrode materials that provide high energy and power densities while also being resilient to electrochemomechanical degradation despite large volume fluctuations [77]. In addition to extensive research on materials discovery, significant advancements have been achieved in the engineering of electrode design for LIBs [78,79]. Uddin et al. [80] and Eom et al. [81] suggested that traditional batteries face constraints such as limited power output and operational lifespan, indicating potential shortcomings in their electrode materials. The electrode manufacturing process begins with the homogenization of battery components—such as active materials, conductive additives, and binders—in a suitable solvent, which influences the properties of the final product. The electrode slurry is then coated onto metal foils (aluminum for positive and copper for negative electrodes) using either simple lab-scale methods like the doctor blade or advanced industrial techniques such as slot-die coating. After coating, the electrodes undergo drying and calendaring to achieve the desired thickness, followed by slitting, winding, packaging, and the final assembly and inspection of the battery cells (refer to Figure 4). Ensuring optimal mass ratios and solvent selection is crucial for enhanced performance attributes [82]. The choice of electrode materials in batteries is critical, influencing energy density, power density, and cycling stability, all of which are essential factors determining the overall performance of batteries [83]. The rising demand for consumer electronics, EVs, and renewable energy has sparked significant interest in organic electrode materials, which provide advantages like sustainability, cost-effectiveness, stability, flexibility, and a lower carbon footprint compared to traditional inorganic electrodes [84]. Organic electrode materials exhibit low discharge potentials and charge–discharge rates, which makes them suitable for cost-effective and environmentally friendly rechargeable ESSs that do not depend on metals like lithium [84]. Current battery technology struggles to meet the energy demands of EVs due to non-linear energy consumption and discharge fluctuations. A potential solution is to pair batteries with supercapacitors that provide additional energy when needed. Alternatively, enhancing electrode materials with short diffusion lengths and high surface area can improve the performance of LIBs [85]. Liu et al. [86] discuss how slurry mixing, coating, drying, and calendaring influence the microstructure and electrochemical performance of electrodes in LIBs, providing critical insights for the advancement of battery technologies in future research. Enhancing the electrochemical performance of electrode materials for LIBs requires effective surface and interface engineering [87]. Croguennec et al. [88] provide a critical overview of inorganic electrode materials for LIBs, categorizing them by reaction mechanism and structural dimensionality. Overcharging or excessively discharging LIBs can cause irreversible damage to the electrodes. Minimizing electrode thickness is proposed as an effective strategy to enhance power density while simultaneously reducing current density and resistance [89]. Commercially successful electrodes often use intercalation materials, which enable reversible ion insertion through one-dimensional or two-dimensional pathways in their crystalline structures. To optimize the deliverable energy of a battery, it is crucial to focus on three main aspects. First, the potential difference between the anode and cathode should be maximized. Second, it is important to minimize the mass and volume of active materials used per electron exchanged. Third, it is critical to prevent the oxidation or reduction in the liquid electrolyte to preserve its integrity [90,91]. Advancing LIB performance through next-generation electrode development is crucial for achieving net-zero emissions. A comprehensive understanding of how electrode architecture and design impact properties and performance is necessary, as current designs often rely on empirical trade-offs. Additionally, selecting optimal manufacturing processes remains challenging due to limited understanding of their benefits and limitations [92]. A critical distinction between batteries and supercapacitors lies in their energy and power density capabilities [93,94,95,96,97]. Batteries are renowned for their high energy density, allowing them to store substantial energy per unit mass. However, they often need to catch up to supercapacitors in delivering swift power output. Additionally, it is widely believed that batteries need to be improved by intrinsic limitations, such as diminished performance in low temperatures and a finite number of charge–discharge cycles. These constraints escalate management costs and shorten their overall lifespan [98]. Hence, there is a clear impetus for developing alternative energy storage solutions that can mitigate the deficiencies of traditional batteries, particularly in scenarios requiring high power delivery and enhanced operational flexibility [99]. Furthermore, Shen et al. [100] stated that integrating supercapacitors alongside batteries in large-scale systems could alleviate the strain on batteries during brief disruptions, potentially extending their operational lifespan. While supercapacitors exhibit considerable potential as alternatives or complementary solutions to conventional batteries, their present energy density remains insufficient in comparison.

### 2.3. Electrolytes and Separators: Functions, Types, and Recent Advancements

In rechargeable LIB systems, electrolytes play a crucial role in facilitating ion movement while preventing electron flow between the anode and cathode during charge and discharge cycles [101,102,103]. These electrolytes come in various forms, including organic liquids, ionic liquids, aqueous solutions, inorganic solids, and polymer composites, each offering unique properties suitable for different battery applications [104,105,106]. A commonly used organic liquid electrolyte employs ethylene carbonate due to its high dielectric constant and ability to dissolve several lithium salts, thus enhancing ionic conductivity [107,108]. Popular lithium salts such as lithium hexafluorophosphate are often dissolved in solvent mixtures containing ethylene carbonate and linear carbonates like diethyl carbonate, dimethyl carbonate, or ethyl methyl carbonate [107,109]. This combination improves thermal stability and reduces viscosity, crucial for efficient Li^+^ conductivity. High Li^+^ conductivity is essential for effective ion transfer, ensuring the battery’s rapid charge–discharge rates and power performance. Thus, selecting the right electrolyte composition is integral to optimizing LIB efficiency and stability [110]. Developing electrolytes with high lithium-ion conductivity, low viscosity, and electrode compatibility is essential for the advancement of LIB technology [111]. Electrolytes and separators play crucial roles in LIB technology, especially in addressing challenges like poor rate capability, volume change, energy density, and safety concerns. Recent advancements focus on enhancing these components to meet demands for low-cost, large-scale energy storage from renewable sources. Tesla’s success with LIBs in EVs highlighted the importance of improving power, range, charging rate, lifetime, and safety for broader commercialization. Simulation and modeling are crucial in advancing LIB technology, aiding material development, and understanding complex electrochemical processes [112,113]. However, ensuring the accuracy of theoretical predictions to real-world conditions is paramount for guiding industrial-scale battery production [114,115]. This holistic perspective emphasizes the significance of accounting for broader system-level implications alongside the nuances of battery chemistry and design [116]. In parallel, separators emerged as pivotal components in battery technology, serving the critical function of preventing short circuits within the battery while facilitating the passage of ions between electrodes. Typically constructed as thin, porous membranes from materials like polyethylene, polypropylene, or ceramic compounds, separators have witnessed notable advancements in recent years [117]. Recent research endeavors have been dedicated to enhancing separator technology, focusing on bolstering mechanical strength, thermal stability, and electrochemical performance. Particularly noteworthy were advancements in ceramic separators, which boasted superior thermal and chemical stability compared to conventional polymer separators. These innovations enhanced battery safety and held promise for applications in high-temperature or high-voltage environments. Recent discussions emphasized advancements in LIB technology, yet Nayak et al. [118] underscored the crucial roles of electrolytes and separators for safety and performance. Ceramic separators offered enhanced protection. Casta et al. [119] thoroughly reviewed the strengths and weaknesses of various separator types, offering insights into potential areas for innovation. Wu et al. [120] and Heimes et al. [121] also stressed their importance, advocating interdisciplinary collaboration to integrate theoretical insights and experimental validation for next-gen solutions. While theoretical modeling, electrolytes, and separators are essential to advancing LIB technology, there are ongoing challenges and areas for improvement in accuracy, practical applicability, safety assurance, and research methodologies. Although extensive research has been conducted, ongoing advancements in material development are essential for further improvement.

### 2.4. Cell Designs and Configurations: Cylindrical, Prismatic, and Pouch Cells

**Figure 5:**
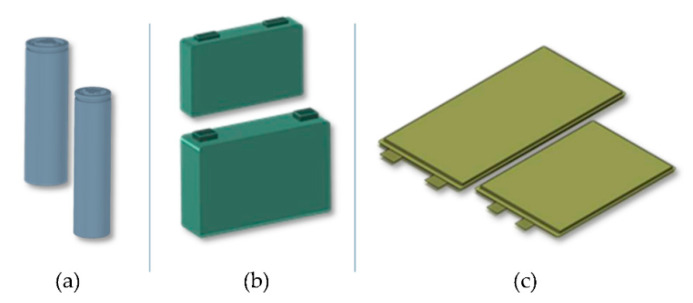
Three kinds of standard cell formats: (**a**) Cylindrical cells, (**b**) Prismatic cells, and (**c**) Pouch cells [122].

Battery cells are a critical component of battery systems. The writers highlight the pressing need for advancements in cell technology by identifying the limitations of the traditional tab design, including safety concerns and efficiency losses, to meet the industry’s evolving demands. Highlighting the urgent need for advancements in cell technology, this analysis shows the challenges and innovations within the industry, with an analysis of Industry trends that complements this examination, emphasizing the increasing popularity of cylindrical cells in the automotive sector and the potential for advancements in battery technology. LIBs come in a variety of shapes that are competing as energy storage solutions for diverse applications. The main designs include cylindrical cells, pouch cells, and prismatic cells. The primary distinctions among these cell designs lie in the structure of the cell casing and the arrangement of the cathode, separators, and the anode [122,123,124,125,126,127,128]. Until several years ago, research into manufacturing processes and cost models was primarily focused on prismatic cells. No specific model addressed the manufacturing costs of cylindrical cells [129]. Figure 5 illustrates the standard cell formats commonly used in the automotive industry. Table 3 shows comparison of the advantages and disadvantages of cylindrical, prismatic, and pouch cells for their application in EVs.

Ciez and Whitacre’s [129] views on cylindrical LIB cell designs and configurations offered a critical examination of the challenges and innovations within the industry. The researchers [130,131,132,133,134] believed that Tesla’s introduction of a new jelly roll design for their 4680 cells represented a proactive step towards addressing these challenges. This nuanced perspective demonstrated an understanding of both the opportunities and challenges inherent in pushing the boundaries of battery design. The writers discussed critical insights into the evolving landscape of battery technology. Brauchle’s [135] analysis underscored the increasing popularity of cylindrical battery cells in the automotive sector, emphasizing the necessity for technological advancements to meet evolving demands. However, a more in-depth examination of underlying trends and comparative advantages would enhance the analysis. While empirical evidence supports their arguments, a critical evaluation of the methodology and potential biases would be beneficial [136]. Frank et al. [137] highlighted the importance of understanding cylindrical cell design principles for LIB advancement, while Sturm et al. [138] emphasized pouch cells’ superior energy density and simpler manufacturing processes, echoing the theme of market accessibility emphasized by Waldmann and colleagues [139]. These perspectives underscore the complexity of battery technology evolution, warranting further research and scrutiny for effective advancement. However, there is a potential need for more comprehensive exploration into the underlying reasons driving industry trends and preferences for specific battery cell designs.

## 3. Advances in Materials and Technologies

### 3.1. Anode Materials: Graphite, Silicon, and Beyond

**Figure 6:**
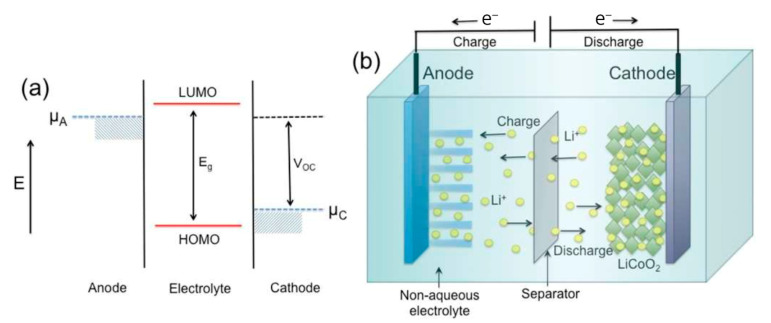
(**a**) A visual representation of the relative energy diagram of electrode potentials and the energy gap in the electrolyte for LIB: In a LIB, the relative energy levels of the electrodes and the electrolyte determine the battery’s voltage and stability. (**b**) A schematic diagram outlining the mechanism of lithium intercalation and de-intercalation within rechargeable LIBs equipped with solid electrodes and liquid electrolyte [160] (reprint with permission from The Royal Society of Chemistry).

For realizing the next-generation rechargeable Li and Li-ion batteries with higher energy densities, longer cycle life, and better safety, improving progressed anode materials is important, as their choice enormously impacts these parameters, primarily battery capacity, cell voltage, and operating voltage. Despite significant research efforts to find alternatives with superior strength and strength density, graphite remains the material of choice in commercial LIBs [140,141]. Beyond the conventional graphite and lithium-titanium-oxide (LTO) anodes, extensive research has developed several high-capacity anode materials to address the increasing demand for rechargeable batteries [142]. The field of high-capacity anode research has been extremely active, with a strong focus on materials such as silicon (Si), silicon oxide (SiO_x_), tin (Sn), and tin oxides (SnO_x_). These materials have garnered enormous attention from the research community due to their promising capacity and performance characteristics. However, the landscape of high-capacity anode materials extends beyond these well-studied ones as various other, sometimes more exotic materials have also been explored in the literature [143]. The energy density of LIBs is closely tied to the electrode materials and their specific capacity. Notably, anode materials present a greater capacity enhancement potential than cathode materials [144,145,146]. Enhancing the energy density of conventional LIBs is essential to meet the evolving requirements of EVs and advanced electronic devices. Silicon, recognized as one of the most promising anode materials, offers significant potential to replace conventional graphite anodes, facilitating the development of high-energy LIBs [147]. The widespread adoption of silicon-based anodes in LIBs is severely impeded by their substantial volume variations during cycling and low electrical conductivity. The most promising strategies to surmount these obstacles and facilitate the practical utilization of silicon-based anodes encompass the judicious design of silicon structures and the synergistic integration of nano-scale silicon with carbon-based materials. A succinct examination of the mechanisms underlying lithium-silicon alloying and cell failure is crucial to comprehend the inherent limitations of silicon-based anodes [148]. A particularly effective approach for near-future commercialization involves combining silicon and graphite. This method capitalizes on existing production facilities, simplifies manufacturing processes, and reduces capital investment, thereby streamlining the path to market readiness [147]. However, combining silicon and graphite presents several challenges, including incompatibility and inadequate material bonding. To overcome these obstacles, various carbonaceous materials have been extensively introduced [147]. Wu et al. delineated the design of various shapes of nanostructured Si, such as hollow nanostructures and nanowires, which effectively mitigate the volume expansion problem during charge and discharge cycles [149]. Additionally, Liang et al. concentrated on developing silicon-based composites, including Si/amorphous carbon, Si/graphene, Si/carbon fiber, and Si/polymer, and examined their electrochemical performance [150]. These reviews primarily focused on the research advancements in nanoscale silicon and silicon-based nanocomposites. However, they often overlooked silicon-based composites’ practical applications and scalability, which are critical for commercial viability [151]. Exploring the evolution of anode materials in LIBs, the research highlights the pivotal role of graphite in revolutionizing battery technology, offering improved energy densities and cycling stability. Despite graphite’s dominance, challenges persist, prompting the investigation of silicon-based materials with higher capacities. This underscores the need for a comprehensive review focusing on its fundamental mechanisms and recent advancements to address existing challenges. Hassoun and Scrosati [152], Li et al. [153], and Zhang et al. [154] provided historical perspectives on the development and dominance of graphite-based anodes in LIBs, highlighting their significant advancements and commercial success. They established a foundation for understanding the evolution of anode materials in battery technology. Ge et al. [155] and Chadha et al. [156] critically evaluated the limitations of graphite anodes and explored the incorporation of silicon-based materials to address these challenges. They introduced silicon as a promising alternative with higher capacities and highlighted its associated issues, such as volume expansion. Zhao and colleagues [157] emphasized the persistent preference for graphite despite ongoing research into alternative anode materials like silicon. They underscored the necessity for a comprehensive review focused specifically on graphite anodes, covering fundamental mechanisms, practical considerations, and recent developments. The selection of materials for anodes is fundamentally influenced by several critical factors, including their crystal structure, physical attributes (specific capacity, electrical conductivity, and mechanical stability), and chemical characteristics (such as intercalation and reversibility). Moreover, the size and shape of these materials play a crucial role in enhancing the performance of LIBs. Initially, the first generation of anodes predominantly featured micrometer-sized particles designed for lithium intercalation and de-intercalation reactions. However, precise control over the material size is essential for optimizing the overall performance of LIBs [158]. Due to increased surface area, Rutile TiO2 nanoparticles (5–15 nm) showed enhanced Li accommodation than micrometer-sized particles [159]. However, besides nanomaterials, miniaturization enhances charge–discharge rates and can trigger undesirable side reactions, impacting battery longevity. Figure 6 describes a relative energy diagram illustrating the electrode potentials and the electrolyte energy gap for a LIB [160]. Part (a) shows the energy diagram, indicating the anode and cathode potentials, energy gap (E_g_), and open-circuit voltage (V_OC_). Part (b) depicts the LIB mechanism, where lithium ions move between anode and cathode during charge–discharge cycles, while the separator prevents short circuits. This demonstrates the fundamental principles essential for LIB functionality and safety.

The use of carbonaceous materials as anode components in LIBs has been extensively advocated in the scientific literature and yielded considerable success. These materials provide higher specific charge capacities and more negative redox potentials than metal oxides, chalcogenides, and polymers [161]. Carbonaceous materials come in various forms, including graphite/graphitized materials and non-graphitized soft and hard carbon, each exhibiting unique structures and electrochemical characteristics. One-dimensional carbon nanostructures, such as carbon nanotubes (CNTs), have been especially noted for their exceptional properties. Their sp^2^ hybridization, high surface area, excellent conductivity, and rapid ion transport make CNTs highly advantageous. Various synthesis methods, including acid oxidation, ball milling, and chemical vapor deposition, can produce single-walled (SWCNT) or multi-walled (MWCNT) structures, depending on the desired morphology [162,163]. Rosenzweig et al. [164] stated that decreasing the particle size of CNT can lead to better strain properties, enhancing electronegativity relative to bulk graphite. In addition, 2D carbon nanostructures such as graphene nanosheets are investigated and utilized extensively as anode materials in LIBs, either independently or in hybrid forms. Two-dimensional carbon nanostructures have single-atomic-layer thickness, aromatic carbon network, high conductivity, and exceptional lithium storage capacity owing to the high surface area, which makes them highly desirable and can accommodate Li ions on both sides of the sheet and at defect sites. Finally, 3D porous carbon nanostructures with different pore sizes emerged as an alternative anode material for LIBs in which carbon network and high surface area are interconnected, leading to facilitation of superconductivity and strain accommodation during charge–discharge cycles [160]. Spinel-structured Li_4_Ti_5_O_12_ over graphite has found its place as an anode material in LIBs, operating at a higher potential (~1.5 V vs. Li/Li^+^). It can be used to avoid the formation of the electronically insulating solid-electrolyte interphase (SEI) layer. Still, it suffers from poor electronic conductivity and moderate Li^+^ diffusion coefficients, which can be mitigated by size reduction and conductive coating despite its high specific capacity and cyclic stability. Moreover, many metal alloys based on Li, namely Li_x_M, are evaluated due to their potential as electrode materials in LIBs (M stands for a secondary electrochemically active or inactive metal used to make structural support and enhance electrical conductivity during lithium intercalation–deintercalation processes) [165,166]. Recent efforts have focused on nanostructured materials to enhance electron and Li^+^ transport. Nevertheless, challenges remain in fully replacing graphite, largely due to complex electrochemical mechanisms. Ongoing research is vital for understanding these processes to develop high-performance, reliable batteries.

In pursuit of improving the performance of LIBs, various anode materials are explored, including alloying (e.g., Si, Sn), conversion (e.g., Fe_2_O_3_), and intercalation (e.g., graphite, Li_4_Ti_5_O_12_). Alloying materials offer high theoretical capacities and utilize abundant resources, although they face challenges such as significant volume changes and safety concerns. Conversion materials similarly present high energy densities but struggle with irreversible capacity loss and poor rate capabilities. In contrast, intercalation materials exhibit excellent cycle life and established manufacturing processes, yet they are limited by their lower theoretical capacities and safety risks at elevated charge rates. Table 4 compares various anode materials for LIBs, detailing their types, mechanisms of anodes in rechargeable LIBs, and the advantages and disadvantages of alloying, conversion, and intercalation-based anode materials [167,168].

### 3.2. Cathode Materials: Transition Metal Oxides, Lithium Iron Phosphate, and High-Voltage Materials

**Figure 7:**
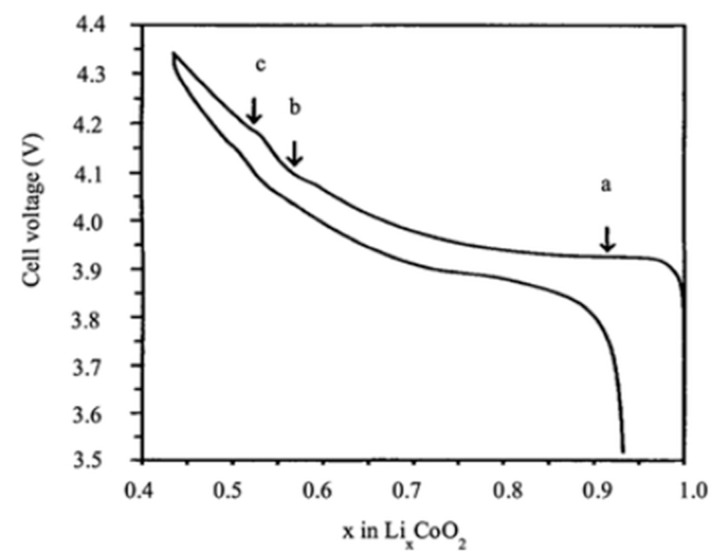
The cell voltage of Li_x_CoO_2_ as a function of the lithium content (x): The voltage profile of Li_x_CoO_2_ exhibits several distinct plateaus and sloping regions, which correspond to different phase transitions that occur during the lithium intercalation/deintercalation process. The plateau *a* corresponds to a first-order transition; plateaus *b* and *c* are two order/disorder transitions that were observed from hexagonal to monoclinic, then from monoclinic to hexagonal [174].

Cathode materials are essential in LIB performance, addressing the evolving demands of applications like electric mobility and renewable energy solutions. Beginning with the historical context of LIB development, there is a need for advancements in cathode materials to meet challenges posed by global warming and increasing EV demands. Transition metal oxides and LFP emerge as prominent cathode materials with distinct characteristics and advantages, addressing stability and capacity concerns while acknowledging limitations for LIB applications. Commercialized LIBs comprise lithium transition metal oxide as a cathode (LiCoO_2_, NCM, NCA, or LiFePO_4_) [169]. To enhance the energy density of LIBs, researchers have implemented several strategies. These include improving cathode active materials, increasing the specific capacity of cathode and anode materials, exploring lithium metal and anode-free battery designs, utilizing solid-state electrolytes, and developing innovative ESSs [170]. Cathode materials with high electrochemical potentials have garnered significant interest and have been thoroughly reviewed in the literature [171,172]. Several cathode materials, including LiMn_2_O_4_, LiFePO_4_, and LiNi_0.8_Co_0.1_Mn_0.1_O_2_, have been extensively researched for large-scale LIBs to replace LiCoO_2_. However, these materials encounter challenges in achieving the high energy density and specific capacity required for various applications at a low cost. Their specific capacities, typically below 250 mAhg^−1^, prove inadequate for some applications [173]. LiCoO_2_ has played a crucial role in the development of rechargeable LIBs. Sony’s innovation in combining LiCoO_2_ as the cathode material with a carbon anode led to the creation of the first commercially successful rechargeable LIB, which operates at ca. 3.7 V vs. Li/Li^+^. From an electrochemical perspective, Li_x_CoO_2_ is highly attractive due to its high theoretical capacity of 274 mAh/g and impressive energy density. However, its performance can be compromised by structural and thermal instability: at high voltages and elevated temperatures, LiCoO_2_ may experience structural changes that impact its stability. Regarding capacity fade, rapid capacity loss can occur with high current rates or deep cycling, limiting the battery’s lifespan. During the charging process, Li_x_CoO_2_ undergoes multiple phase transitions as lithium ions are de-intercalated. Figure 7 illustrates the typical charge–discharge behavior of Li_x_CoO_2_ about the lithium content (x) [174].

**Figure 8:**
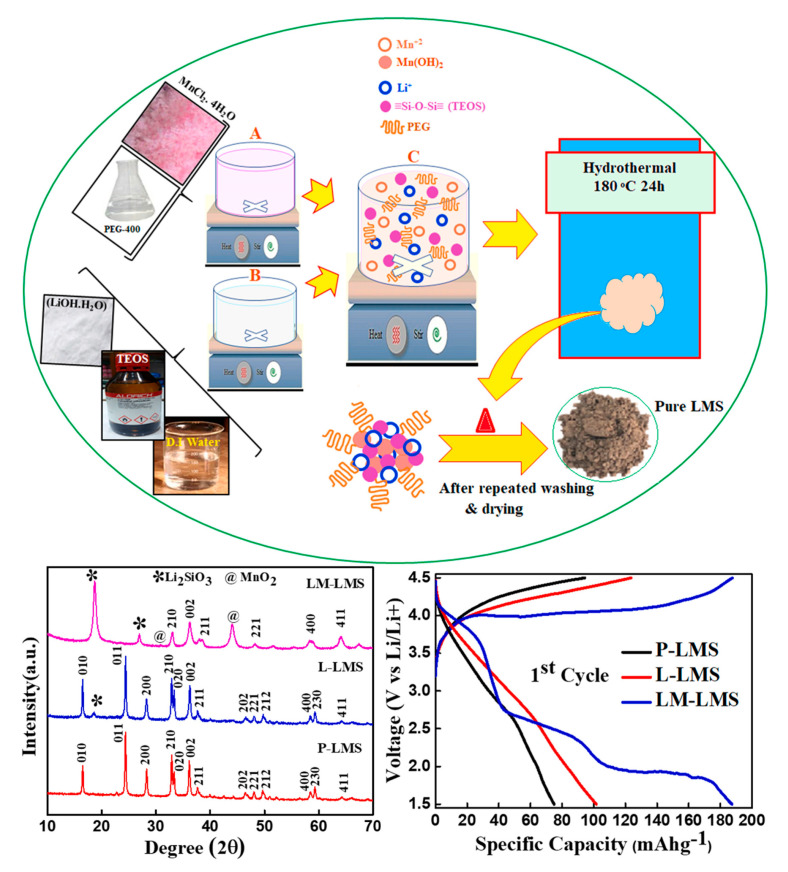
The synthesis process of Li_2_MnSiO_4_ utilizes the hydrothermal method, resulting in the production of high-quality cathodes [217] (reproduced with permission from Elsevier).

Among these, 3D transition metal-doped spinel cathode materials stand out as high-voltage materials with superior specific energy compared to their parent compound, LiMn_2_O_4_. These materials are considered promising candidates for next-generation LIB technology [175]. Li-stoichiometric mixed metal oxides and Li-excess Mn-based oxides have been considered the most promising families of cathode materials for large-scale LIBs with high energy density used for transportation [176]. Layered lithium transition metal oxide cathode materials have also been extensively studied, highlighting their potential to improve battery performance [177,178,179]. In another paper, the authors investigated a LIB based on a copper-supported graphene nanoflake anode and a LFP cathode, which exhibits auspicious performance in terms of cycling life and speed capability [180]. Lithium polyanion has been investigated as a cathode material for LIBs. LiFePO_4_/C, as a cathode material, has been applied to EVs [181]. Developing high-energy-density LIBs is a global priority to meet diverse application needs. One effective strategy to boost LIB energy density is increasing the output voltage, which largely depends on cathode materials. The most promising high-voltage (>4 V vs. Li/Li^+^) LIB cathode categories are lithium-rich layered oxides, nickel-rich layered oxides, spinel oxides, and high-voltage polyanionic compounds. However, these cathodes still face significant challenges in improving output voltage, maintaining high capacity, fast rate capability, and long service life [182]. Table 5 presents a comprehensive overview of various innovative approaches aimed at enhancing the performance of cathode materials in LIBs. Each method is described in detail, highlighting the underlying mechanisms and their respective advantages [167,183,184,185]. Kraytsberg et al. thoroughly reviewed the current status and future directions in high-voltage cathode materials for LIBs. Their analysis underscores these materials’ pivotal role in enhancing lithium-ion cells’ energy density. High-voltage cathodes, which operate above 4.5 V versus Li/Li^+^, offer substantial improvements in battery energy density compared to conventional cathodes [186]. According to F.M. Nizamuddin Khan et al. [187], increasing cathode thickness significantly enhances cell energy density. Additionally, there is significant interest in identifying new cathode materials that balance performance and environmental requirements [174]. Cathode materials’ stability and degradation rate impact cell aging [188,189]. Goodenough [190] provided broad overviews of cathode materials, underscoring their significance in determining LIB performance and addressing evolving industry demands. The conventional cathode materials for LIBs, primarily insertion-type metal oxides like layered LiMO_2_ and spinel oxides, offered stable electrochemical performance but faced limitations such as voltage constraints and capacity degradation over cycling [191]. Li, Meng, and Jin [192] and Nan et al. [193] represented challenges of current cathode materials, proposing solutions to enhance energy density, safety, and cost efficiency. Ma et al. [194] spotlighted (LiFePO_4_) for its remarkable thermal stability and safety, enriching the discourse on cathode material selection for LIBs. Their insights underscored the diverse strategies in cathode material advancement, augmenting comprehension in the field. However, a deeper examination of these perspectives could offer a more holistic understanding. As previously mentioned, LiCoO_2_, as the first commercially used positive electrode material introduced by Sony in 1991, marked a significant milestone in the development of LIBs, demonstrating high theoretical capacity and energy density [195]. However, structural and thermal instability at high voltage and temperature hinder the performance of these cathode materials. Additionally, they suffer from rapid capacity fade during high current rates or deep cycling. During the charging process, numerous phase transitions can occur at different stages, further impacting their stability and efficiency. LiCo_1−x_Ni_x_O_2_ has been proposed as an alternative to LiCoO_2_ [196]. In these rechargeable batteries, LiNiO_2_ combines the high capacity associated with LiNiO_2_ and the stability of LiCoO_2_. This solid solution can be formed across the entire x range, with cell volume and lattice parameters increasing linearly while c/a decreases following Vegard’s law with Ni substitution for Co [197]. It has been demonstrated that higher Co content in these batteries enhances structural stability and suppresses non-stoichiometry. The optimal performance of LiCo_1−x_Ni_x_O_2_ is achieved within a special range (from 0.1 to 0.3) [198,199]. LiMnO_2_ is a promising cathode material that has gained attention recently due to its lower toxicity and reduced cost compared to cobalt and nickel oxides. Layered LiMnO_2_ can be synthesized using the ion exchange method [200]. The layered structures in these LIB-based batteries are thermodynamically unstable due to the Jahn–Teller effect, resulting in orthorhombic or monoclinic structures. It is reported that monoclinic LiMnO_2_ is less stable than spinel and orthorhombic LiMnO_2_ because of antiferromagnetic interactions between Mn^3+^ ions. Moreover, LiNi_0.5_Mn_0.5_O_2_ is highly suggested, with a theoretical capacity of 280 mAh/g. Ni^2+^ undergoes oxidation to Ni^4+^ in two stages in this type of battery, while Mn^4+^ remains electrochemically inactive. In addition, LiNi_x_CoyMn_1−x−y_O_2_ is a mixed transition metal oxide family introduced to deal with the shortcomings of individual oxides, such as stability, safety, and specific capacity [201]. In addition, phosphate-based cathode materials, specifically Polyanionic olivine LiMPO_4_ (M = Fe, Mn, Co, or Ni), have been widely suggested due to their considerable potential in the case of LIB applications. LiMPO_4_ has an olivine structure in which oxygen ions form a hexagonal close-packed framework with Li occupying the octahedral M1 site, forming a tunnel along the [133] direction [202]. The structural characteristics of certain materials significantly impact their performance as cathode materials for rechargeable batteries. For instance, some materials exhibit a zigzag chain of corner-shared octahedra connected to phosphate tetrahedra through transition metal ions (such as Fe, Ni, Mn, or Co) located at the M_2_ site. The MO_6_ octahedra and PO_4_ tetrahedra share edges, leading to strong P–O covalent bonds. These bonds contribute to high thermodynamic stability, effectively preventing oxygen evolution at elevated temperatures, a common issue in materials like LiCoO_2_ and LiNiO_2_. It can also result in a lower redox energy of the metal’s 3D orbital relative to the Fermi level. The literature proves that LiMnPO_4_ has higher discharge voltage and energy density than LiFePO_4_, making it an important choice, especially in applications like the automotive industry [203]. Despite its potential, LiMnPO_4_ encounters significant challenges related to cyclic stability and rate capability, primarily due to its poor kinetics and low ionic and electronic conductivity. Researchers have developed silicate-based cathode materials to overcome these issues, which represent a major advancement in high-energy cathode technology. Specifically, orthosilicate cathode materials, such as Li_2_MSiO_4_ (M = Fe, Mn), have shown promise. These materials stand out due to their excellent electrochemical performance, enhanced safety, availability of raw materials, and cost effectiveness. Consequently, orthosilicate materials are highly advantageous for developing more efficient and reliable LIBs [204]. Figure 8 shows the process of Li_2_MnSiO_4_ synthesis as a cathode, Table 6 summarizes the key properties and applications of some common LIB cathode chemistries, and Table 7 shows the recent advances in the case of cathode materials.

**Table 5:** Approaches to enhance cathode material performance.

Approach	Description	Key Benefits
Doping	Introducing aliovalent cations (e.g., Mg, Al) into the cathode lattice alters electronic/ionic conductivity and creates defects (e.g., oxygen vacancies).	Enhances Li-ion diffusivity, improves rate capability, stabilizes structure.
Coating	Application of a thin layer (e.g., Al_2_O_3_, TiO_2_, carbon) on the cathode surface to prevent side reactions and enhance cyclability.	Reduces interfacial impedance, protects against electrolyte decomposition.
Single Crystal Growth	Growth of single crystals to eliminate grain boundaries that impede diffusion, enhancing structural stability and performance.	Increases rate capability, energy density, and cycle life.
1-D Fibrous Electrodes	Use of nanowires or nanotubes in cathode construction to increase surface area and facilitate Li-ion transport.	Improves electrode-electrolyte contact, enhances flexibility and kinetics.
Electrolyte Optimization	Adjusting electrolyte composition for better ionic conductivity and compatibility with cathodes, reducing side reactions.	Improves interfacial stability and overall battery performance.
Surface Modification	Techniques such as fluorination that adjust the surface chemistry of cathodes to optimize performance.	Creates stable SEI layer, enhances interfacial stability and reactivity.

**Table 6:** The critical properties of standard LIB cathode chemistries.

Cathode	Energy Density	Power Density	Safety	Cost	Key Applications	Ref.
NMC (Ni-Mn-Co Oxide)	High	High	Moderate	Moderate	- EVs - Consumer electronics	[205,206]
(LFP) LiFePO_4_)	Moderate	High	High	Low	- Electrical grid - Storage - Power tools - Electric bikes	[207,208]
NCA (Ni-Co-Al Oxide)	Very High	High	Moderate	High	- Premium EVs	[209]
LMO	Moderate	High	Moderate	Moderate	- Consumer electronics such as smartphones, laptops, and tablets	[210]
LCO	High	Moderate	Moderate	High	- Portable consumer electronics	[208]

**Table 7:** Recent advances in the case of cathode materials.

Cathode	Energy Density	Power Density	Safety	Ref.
LiNiO_2_ and LiCoO_2_ (with Mn and Mg enhancement)	Chemically	- Different performances reported	Not Reported	[174]
2D covalent organic framework	Organically	- Outstanding capacity retention (~81% after 1000 cycles at 1.54 A/g)	Not Reported	[211]
Li-rich Mn-based cathode	Chemically	- Coulombic efficiency (ICE) of 84.1% to 100.7% - Very high specific capacity of 330 mAh/g at 0.1 °C	Formation of microcracks Reduced ionic/electrical conductivities Partial electrochemical insulation Structural inhomogeneity	[212]
Surface-doped cathode materials	Chemically	- Enhanced structural stability, Li-ion diffusion, and electrochemical properties	Lack of detailed understanding of mechanisms and effectiveness	[213]
Hetero-structured cathode design for sodium-ion batteries	Combination of O_3_NaNi_0.5_Mn_0.5_O_2_ and P_2_Na_2_/_3_MnO_2_ coating	- Significant reversible capacity - Outstanding rate capability - Enhanced cycling stability - Enhanced reversibility - Enhanced stability - Improved electrochemical kinetics	- Intricate phase transitions - Intense electrochemical degradation and corrosion during cycling	[214]
The atomic arrangements in de-lithiation-driven complex phase boundaries in high-nickel-content layered cathodes	Deep learning aided super resolution electron microscopy	- Improved understanding of phase transformations in high-Ni cathodes	- Challenging to investigate atomic configurations of complex phase boundaries and transitions	[215]
2D covalent organic framework	Electrochemical characterization of δ-MnO_2_ NDs/Zn ZIBs/ZICs	- Considerable specific capacity - Impressive rate capacity - Satisfactory cycle life with 86.9% capacity retention after 5000 cycles	- Poor cyclability and low rate - Capability of traditional MnO_2_ cathodes	[216]

### 3.3. Solid Electrolytes and Their Potential for Enhancing Safety and Energy Density

Solid electrolytes (SEs) facilitate ion conduction while remaining in a solid phase. Often consisting of a polymer matrix, these electrolytes enable the efficient transport of ions, such as lithium ions in LIBS. SEs are integral to battery technology advancements, particularly in all-solid-state batteries, which serve as liquid electrolyte replacements. Their use offers numerous benefits, including increased safety, superior mechanical strength, and the potential for higher energy density. As such, solid electrolytes are fundamental in the progression of advanced battery systems, contributing to enhanced performance and reliability. Wang and Zhong [218] presented a comprehensive analysis of solid electrolytes, mainly focusing on SEs, and their potential to improve the safety and energy density of batteries. It effectively highlighted the advantages of SEs, such as their flexibility and mechanical integrity, which made them suitable for high energy density and all-solid-state battery designs. Moreover, the absence of organic carbonate plasticizers in SEs was emphasized as a significant factor contributing to enhanced battery safety, particularly in lithium metal applications. However, Chen et al. [219] also critically examined the limitations of SEs, such as their relatively low lithium transference numbers and limited ionic conductivity, attributed to strong coordination between the polymer backbone and lithium ions. Despite these challenges, Wang and colleagues [220] offered insights into various strategies for improving SE performance, including modifying polymer architectures and incorporating ion-diffusion facilitating units or ionic liquids/solvents as plasticizers. This balanced analysis provided a clear understanding of the current state of solid electrolytes and the ongoing efforts to overcome their limitations for next-generation battery technologies. Furthermore, as battery technologies continue to evolve, there is a need to develop solid electrolytes that can meet the increasingly demanding requirements of emerging applications, such as EVs and grid energy storage. This includes achieving higher energy densities, improving cycling stability, and ensuring scalability and cost-effectiveness of production processes. Many electrodes have been introduced in the literature. Typical solid electrolytes demonstrate excellent chemical and electrochemical stability, strong mechanical properties, and high oxidation voltage. For instance, perovskite inorganic solid electrolytes with the crystal structure of ABO_3_ (where A can be Ca, Sr, or La, and B can be Al or Ti) are extensively employed due to their ionic conductivity, which can be tailored by substituting different ions at the A and B sites [221]. In these electrolytes, ions in the perovskite structure move along adjacent A site vacancies and pass through a constriction formed by four adjacent oxygen ions. Introducing a sizeable rare-earth metal ion at the A site enhances ion transport. Li_3x_La_2/3_₋_x_TiO_3_, for instance, can be synthesized by occupying the A site with Li or La, resulting in high ionic conductivity of 10^−3^ S·cm^−1^ at 25 °C. However, for LIBs, the instability of Ti^4+^ concerning Li makes it unsuitable [222]. AM_2_(PO_4_)_3_ (A = Li, Na; M = Ti, Ge, Zr) demonstrated to have the most appropriate lattice size for Li^+^ conduction and with the original formula of NASICON-type inorganic solid electrolytes where ions transport in a 3D M_2_(PO_4_)_3_−framework composed of MO_6_ octahedra and PO_4_ tetrahedra [223]. AM_2_(PO_4_)_3_ commonly has high porosity, which is not preferable for ion conduction. In addition, partially substituting Ti^4+^ with Al^3+^, Li_1+x_Al_x_Ti_2−x_(PO_4_)_3_ was demonstrated to provide high ionic conductivity due to increased Li^+^ density in the framework and spatial migration with lower activation energies [224].

### 3.4. Nanostructured Materials and Composites for Improved Performance

Nanostructured materials can be categorized into several types: nanoparticles, nanowires, nanotubes, nanofilms, and nanocomposites. These materials can be synthesized from various substances, such as metals, ceramics, polymers, and semiconductors. They have applications across various fields, including electronics, photonics, catalysis, biomedical engineering, and energy storage and conversion. Koo, Pilato, and Wissler [225] provided a compelling argument for the crucial role of nanostructured materials and composites in addressing pressing global energy challenges, especially in the transition toward renewable energy sources. Liu et al. [226] emphasized the urgency of mitigating environmental concerns associated with fossil fuel usage, highlighting the potential of renewable energy technologies such as solar, wind, and hydroelectric power to offer sustainable alternatives. Moreover, Ibrahim et al. [227] effectively emphasized the significance of continuous research and development in material science to drive innovation and meet the evolving global energy demands. The pivotal role of nanostructured materials and composites in addressing global energy challenges underscores the urgency of transitioning toward renewable energy sources. This highlights the importance of ongoing research and development in material science to foster innovation and tackle these pressing issues. However, while Guo et al. [228] convincingly discussed the advantages of incorporating nanostructured materials into polymer matrices for energy applications, it could benefit from providing more concrete examples or case studies to illustrate these applications in practice. Concrete examples would enhance the reader’s understanding and lend credibility to the arguments presented. Additionally, while Balu et al. [229] briefly mentioned challenges associated with nanostructured materials, such as compatibility issues and manufacturing optimization, a more in-depth exploration of these challenges and potential mitigation strategies would enrich the discussion. Furthermore, a more balanced perspective on the advantages and limitations of nanostructured materials could strengthen the writer’s views. Overall, while these studies effectively highlight the transformative potential of nanostructured materials in advancing renewable energy technologies, incorporating specific examples, representing challenges, and maintaining a balanced perspective would further enhance the credibility and impact of the writer’s views. Nanostructured materials have drawn considerable interest as potential electrode materials for LIBs due to their distinctive properties stemming from their small size and unique structural features. These materials, such as nanostructured silicon (Si) anodes, provide benefits like improved electrical connectivity, faster ion transport, and minimized stress and strain during cycling. Various synthesis techniques, including gas-phase reactions and catalyzed vapor–liquid–solid (VLS) growth, contribute to these nanostructures, ultimately enhancing storage capacity and stability [230]. Nanocoating, nanorods, and mesoporous materials have been found to enhance LIB performance by increasing the contact area between the electrode and electrolyte and reducing ion diffusion paths [231]. For example, graphite-like carbon coatings on Si anodes have been investigated to alleviate volume changes [232], and composites such as Li_4_Ti_5_O_12_/Si have been developed to maintain stable cycling even at high currents [233]. In cathode research, optimizing the synthesis of nanostructured LiCoPO_4_ by varying Co^2+^ sources and reaction conditions [234] and improving the charge–discharge performance of Li_2+x_Mn_1−x/2_TiO_4_ by increasing lithium content [235] have shown promising results. Innovative designs for anodes include Si-coated carbon nanofiber core shells [236], ordered mesoporous carbon nanospheres [237], and hydrothermal finger-like Co_3_O_4_ nanorods [238]. Zhao et al. [239] delved into the role of nanostructured materials in enhancing LIB performance, highlighting the advantages of nanoparticles, nanotubes, and nanosheets in improving energy density, power density, and cycling stability. They reviewed the synthesis, characterization, and electrochemical performance of various nanostructured anode and cathode materials such as silicon, tin, and transition metal oxides. Implementing these nanostructured materials addresses issues common in traditional LIB materials, such as volume expansion, low conductivity, and capacity fading. The review emphasizes the importance of rational design and optimization of nanostructured materials to achieve high-performance LIBs suitable for applications ranging from portable electronics to EVs. Wang et al. [240] focused on the development and evaluation of nanostructured silicon–carbon (Si-C) composite materials as anodes for LIBs. These composites, produced by dispersing nanocrystalline silicon in a carbon aerogel followed by carbonization, demonstrated a high reversible lithium storage capacity of 1450 mAh/g, significantly surpassing traditional carbon anodes. The Si-C anodes also exhibited good cyclability, effectively addressing the volume expansion issue typical of silicon-based anodes. This performance is attributed to the uniform dispersion of nanoscale silicon within the carbon matrix, which buffers volume changes during lithiation and de-lithiation processes, thereby maintaining electrode integrity and enhancing cycle life. The researchers propose that nanostructured Si-C composites represent a promising anode material for next-generation LIBs, combining high capacity with stability. In their critical assessment, Komar et al. [241] discussed nanostructured materials’ progress and future directions for energy device applications. They examined the technical benefits, challenges, and potential uses of various nanomaterials in energy storage and conservation, including metal oxides, carbon nanomaterials, and MOFs. Common themes across the research include the necessity of nanostructured materials in advanced battery technologies, the advantages of nanostructured silicon anodes, and the broader challenges in energy storage beyond nanostructures. Emphasis is placed on synthesis approaches for nanostructured materials, in situ characterization techniques, and the potential of metal oxides, carbon nanomaterials, and MOFs in energy storage applications. These studies highlight advancements in electrode materials for LIBs and supercapacitors using nanostructured materials. They underscore the importance of interdisciplinary research efforts and optimization strategies to overcome challenges and maximize the potential of nanomaterials for practical energy device applications. In conclusion, the research underscores the vital role of nanostructured materials in advancing energy storage technologies and calls for sustained research and development in this rapidly evolving field.

## 4. Performance Metrics and Characterization Techniques

### 4.1. Definitions and Measurements of Energy Density and Specific Capacity

LIBs have revolutionized the world of portable electronics, EVs, and ESSs due to their high energy density, long cycle life, and reliability. As the demand for more efficient and sustainable energy solutions grows, understanding the fundamental concepts of energy density and specific capacity becomes crucial in optimizing battery performance [242,243]. These two parameters, energy density and specific capacity, are essential in determining how much energy a battery can store and deliver for a given weight or volume, which directly affects the applications where such batteries can be employed [244,245,246]. For LIBs, higher energy density translates to longer-lasting energy supply without significantly increasing the weight or size of the battery. This characteristic is particularly critical for industries such as EVs, where reducing battery weight while maximizing range is a key design challenge [247,248]. Energy density is a key performance metric for batteries, referring to the amount of energy stored per unit volume or mass, typically measured in watt hours per liter (Wh/L) for volumetric energy density and watt hours per kilogram (Wh/kg) for gravimetric energy density. The total energy stored in the battery is divided by its total mass to measure the gravimetric energy density. The energy density of LIBs can vary depending on the specific composition and design, including factors such as the electrode materials, electrolyte type, and overall cell architecture. The most effective approach to improve the energy density of batteries is to develop and optimize high-capacity electrode materials. The total energy stored in the battery is divided by its total mass to measure the gravimetric energy density. Current LIBs typically range in energy densities, but enhancements are sought for applications like EVs and grid storage. For example, to match the driving range of gasoline-powered vehicles, LIBs with energy densities exceeding 350 Wh/kg are required [249]. Currently, EV ranges typically span from 250 to 350 km per charge. Notably, the Tesla Model S and Nissan Leaf achieve ranges of 500 km and 364 km, respectively [250]. New redox chemistries should accompany a significant increase in energy density, typically occurring between charge-carrier ions and host materials. Choi and Aurbach [251] highlighted the limitation of intercalation-based materials due to their low crystallographic sites in the case of storing charge-carrier ions that lead to the limited energy density of LIBs. Over the past two decades, numerous researchers have concentrated on energy density. Hu et al. [252] introduced a photo-accelerated rechargeable LIB using heterojunction nanoarrays (SnO_2_/TiO_2_) as a multifunctional anode. They demonstrated a considerable enhancement in the specific capacity from 1.91 to 3.47 mAh/cm^2^. They also reported that this method can be used to break the limitations related to the energy density of LIBs for solar-based applications. Many scholars are also concerned with novel cathodes to enhance energy density. Weaving et al. [253] evaluated LiNi_1−x−y_Co_x_Al_y_O_2_ as a cathode and a graphitic carbon anode in many alternative high-energy density Li-ion cell applications. They demonstrated a higher energy density and specific capacity. To accurately determine the particular capacity of LIBs, several steps must be taken into account to specify the amount of energy stored per unit mass of the battery. Various methods can be employed for this measurement, including charge–discharge testing, energy measurement, mass measurement, and calculation-based approaches. Recently, lithium-rich cobalt manganese (LRCM) has been highly suggested due to its outstanding properties, including higher specific capacity and high energy density with the lowest costs; however, because of the complexity of crystal structures and reaction mechanisms, LRCM applications are minimal [173]. As also proven by Abdel-Ghany et al. [254], lithium-rich layered oxides can be appropriately used for LIBs because of their higher capacities than the available cathodes. They showed that coating with AlF_3_ is an effective surface modification technique for enhancing the stability of the lithium-rich layered phase, thereby improving the electrochemical properties of LIBs. The low electronic conductivity and increasing interface charge transfer resistance due to side reactions during cycling have been identified as significant drawbacks of LIBs. Addressing these issues, Li et al. [255] improved the performance of a lithium-rich manganese-based material, specifically Li_1.2_Mn_0.54_Co_0_._13_Ni_0.13_O_2_, by employing a novel method based on Co and Na_x_CoO_2_-coating techniques. Many other researchers have also concentrated on lithium-rich manganese-based materials (LRCMs) due to their high energy density, recognizing their potential as next-generation cathode materials with high specific capacities [173,256,257,258,259]. Many approaches, such as surface engineering, elemental doping, and composition optimization, have received much attention and have shown to have vast research potential [173]. On the other hand, specific capacity (measured in mAh/g) refers to the amount of electric charge that can be stored by the active materials in the electrodes of a battery, per unit mass. Specific capacity is generally measured by charging and discharging the battery under controlled conditions, then calculating the amount of charge stored or released [260,261]. It plays a critical role in determining the overall energy capacity of the battery. For LIBs, specific capacity is mainly determined by the nature of the electrode materials used. For instance, traditional graphite anodes have a theoretical specific capacity of 372 mAh/g, whereas newer materials like silicon anodes offer significantly higher theoretical capacities, often exceeding 2000 mAh/g. In LIBs, researchers are constantly exploring materials with higher specific capacities, such as silicon anodes or sulfur cathodes, to enhance overall battery performance [262,263]. However, silicon anodes face challenges such as volumetric expansion during charging, which can degrade the battery over time. Similarly, cathode materials like LiFePO_4_ are valued for their stability and safety, but they have lower specific capacities compared to newer materials like nickel–cobalt–manganese or nickel–cobalt–aluminum oxides. These newer materials offer higher specific capacities, leading to more efficient energy storage, but may require more complex manufacturing processes and have higher cost. Energy density and specific capacity are fundamental parameters that influence the design, performance, and application of LIBs. Understanding these concepts enables engineers and researchers to innovate and improve upon current battery technologies, pushing the boundaries of energy storage capacity while maintaining safety and longevity. As the global demand for cleaner, more efficient energy sources continues to grow, the need for batteries with higher energy densities and specific capacities becomes increasingly pressing. Advances in materials science, particularly in anode and cathode development, hold the key to unlocking the next generation of LIBs, potentially transforming industries from transportation to renewable energy storage.

### 4.2. Cycling Stability and Calendar Life: Factors Affecting Battery Degradation

LIBs are intricate systems with distinct degradation mechanisms. The primary scientific challenge in battery studies is understanding and modeling these aging processes. Consequently, investigating battery degradation is of paramount importance. Capacity and energy fade arise from a multitude of complex reactions. This degradation is a pivotal issue in battery research, influenced by factors such as battery design, manufacturing, and usage patterns. As batteries age, both their energy storage capacity and power output decline. Battery lifespan can be categorized into two components: calendar life and cycle life. Calendar life relates to the degradation that occurs during storage without charge–discharge cycles, while cycle life pertains to the wear and tear from repeated charging and discharging, as commonly seen in vehicle applications [264]. The design, production, and application affect the degradation of the battery. The factors affecting each part are discussed in Table 8.

The degradation of LIBs throughout their lifespan is an inevitable outcome of the chemical, electrochemical, and mechanical processes within them. These processes, influenced by factors such as battery design, materials, and operational conditions, result in performance degradation commonly referred to as aging. Aging is typically measured using battery health indicators such as capacity fade and resistance growth [287]. The first type of aging, calendar aging, occurs when the battery is not connected to any load for a specific period. The second type, cycling aging, happens during charging or discharging cycles [288,289]. The primary degradation mechanism in calendar and cycling aging is forming and growing a solid electrolyte interphase (SEI) layer at the graphite anode. This SEI layer acts as a protective barrier against corrosion. However, over time, the growth of the SEI layer leads to the loss of lithium ions, electrolyte decomposition, and graphite exfoliation. Lithium plating is another significant degradation mechanism, which occurs predominantly during cycling aging at the negative electrode. This mechanism causes a shift from pseudo-linear to nonlinear behavior in capacity fade over time [290]. LIBs have seen extensive use in new energy vehicles. However, the battery life of LIBs often needs to meet user expectations, hindering the broader adoption of EVs. Therefore, understanding the mechanisms behind battery aging and degradation is crucial for optimizing battery design and management, particularly for vehicle applications. Several critical factors need to be considered in the design process of LIBs. Among these are the aging mechanisms and degradation models at the cell level, which are influenced by critical parameters such as the thickness and porosity of the active materials. For vehicle applications, key degradation factors include capacity fade, power fade, loss of lithium-ion inventory, loss of active materials, increased internal resistance, and loss of electrolytes. The internal physical and chemical side reactions that cause these degradative effects are often complex and interconnected. Degradation mechanisms such as graphite exfoliation, metal dissolution, loss of active materials, SEI film formation, lithium deposition, and electrolyte loss must be thoroughly investigated to mitigate battery degradation, as previously discussed for general LIBs. Guo and colleagues [291] underscored that choosing a LIB for vehicle applications necessitates meticulous consideration of multiple factors. The performance characteristics of an electric storage and the chemical composition of its components heavily influence LIB. Consequently, industries must prioritize the essential properties of LIBs, as these can significantly impact the quality and service life of the traction power source. Several critical factors affect the lifespan of a LIB, including discharge depth, battery temperature, charge–discharge currents, and the range of battery charge. However, as noted by Anseán et al. [292], the degradation of LIBs involves multiple stages that often interact concurrently with varying degrees of intensity, making elucidating battery aging mechanisms a challenging endeavor. Recent advancements in identifying battery degradation have introduced in situ incremental capacity (IC) and peak area (PA) analysis methods. Being non-destructive and capable of real-time application, these techniques can be feasibly integrated into on-board battery management systems (BMSs). Nonetheless, understanding and applying IC and PA techniques require a combination of electrochemical and material science expertise. Galatro et al. [290] conducted a study addressing potential issues associated with data-based degradation models for LIBs. Initially, they combined calendar and cycling tests on various types of LIBs. They demonstrated a strong correlation between the degradation rate (α) and stress factors through linear regression analysis. Additionally, they proposed a methodology for developing degradation-based performance models. This methodology involves selecting appropriate trajectory equations, developing coupled kinetic models, and integrating terms for both calendar and cycling modes.

### 4.3. Electrochemical Characterization Techniques for LIBs: Cyclic Voltammetry, Impedance Spectroscopy, and In Situ/In Operando Methods

LIBs stand out as exceptional energy storage devices across various applications. Given the rising demand for high-performance batteries, the role of electrochemical analysis, specifically in understanding electrode reactions, is becoming indispensable. These aspects can illuminate the intricate relationships between a battery’s design, operational efficiency, and overall performance, ultimately guiding advancements in battery technology [293]. To study these processes, various sophisticated electrochemical analysis methods are used. These techniques can determine the precise voltage points of oxidation and reduction, identify impurities, and confirm the completeness of reactions. Such analyses are crucial in fields like energy storage systems, where LIBs play a pivotal role [294,295]. Numerous characterization methods have been introduced in the literature to evaluate the electrochemical mechanisms and structure–property relationships in lithium-based batteries. Commonly employed electrochemical techniques include cyclic voltammetry (CV), linear sweep voltammetry (LSV), stripping voltammetry (SV), and chronoamperometry (CA). CV has proven to be a vital tool for gaining crucial thermodynamic and kinetic insights into redox processes. However, CV application has often been limited to general information and needs to be appropriately emphasized [293,296,297]. Table 9 provides an overview of the applications of voltammetry in the study of LIBs. CV, based on LSV, measures current as voltage is linearly varied over time. The scan rate is crucial in determining electron transfer rates and material reactivity. The interpretation of CV results is frequently incomplete or subject to debate. Despite this, CV has historically been used as an analytical approach among electrochemical methods.

CV finds extensive application across various domains of electrochemistry, facilitating the analysis of electrochemical redox reactions of active materials by documenting the current response to voltage changes. CV presents several advantages over other measurement methods, including the following [293,298]:

1- The ability to measure under varying test conditions;
2- The ability to ascertain whether the chemical reactions of the reactants are reversible or irreversible;
3- The capability to identify the specific voltage at which oxidation or reduction reactions occur;
4- The feasibility of conducting quantitative investigations for substances with unknown concentrations.

**Table 9:** Applications of Voltammetry for LIBs.

Application	Description	Ref.
Sweep Rate Voltammetry	This technique is effective for examining the pseudo-capacitive properties of cathode materials, such as LiF-MO, where a large portion of the capacity results from surface reactions. These materials demonstrate exceptional power capabilities, maintaining a high discharge capacity even at elevated current densities. It is also used to study anode materials’ mechanisms, calculating contributions of capacitive vs. intercalation reactions, affected by particle size and sweep rate.	[293,295,299,300]
Estimation of Diffusion Coefficients	The method is employed to determine the diffusion coefficients for both cathodes and anodes. It is applied to various cathode structures, including layered and spinel types. This analysis helps in understanding Li^+^ movement during phase transitions, with the technique producing results comparable to other methods, though careful interpretation is required for phase-transitioning materials.	[293,301,302]
Stepwise Voltage Window Technique	This approach is useful for analyzing redox reactions, particularly those involving anionic behavior in Li-rich cathode materials. By incrementally increasing the voltage limits during testing, researchers can uncover unique behaviors and changes in charge capacity, allowing for a clearer understanding of contribution from anionic oxidation processes throughout charge–discharge cycles.	[293,303]

In addition, impedance spectroscopy is also introduced as a powerful electrochemical method used to characterize LIBs. This method applies a small amplitude alternating current (AC) signal across the LIBs and measures the resulting voltage response. By analyzing the impedance spectra obtained, spectroscopy can provide valuable insights into the electrochemical processes occurring within the battery, such as ion transport phenomena, charge transfer kinetics, and electrode/electrolyte interfaces. It can also provide valuable results to assess the performance, degradation mechanisms, and overall electrochemical behavior of LIBs under various operating conditions. In addition, the characterization of impedance has many benefits in the case of identifying battery operational limits, estimating performance, and monitoring state of health (SOH) and state of function (SOF) [304,305]. It is known that commercial LIBs consist of complex systems contributing to impedance sources (e.g., resistive, capacitive, and inductive elements) [306,307]. Electrochemical impedance spectroscopy (EIS) has been extensively adopted as a non-destructive method for characterizing LIBs. This technique provides valuable insights into quality assurance, state estimation, internal temperature monitoring, and second-life applications. EIS operates by applying sinusoidal current or voltage signals across a range of frequencies, allowing for the generation of impedance spectra. These spectra reveal critical information about the capacitance resistance, resistance, and inductance of the cell components and their processes [308]. Interpreting impedance spectra can be very promising in understanding battery behavior under different operating conditions and facilitates battery management and control strategies [309,310]. EIS systems offer numerous advantages. EIS can isolate and quantify bulk cell resistance (Rb), interface layer (RSEI), charge transfer reaction (Rct), and diffusion process (W) in one experiment. EIS spectrum measurement does not require battery cell isolation, which is very beneficial. EIS has high accuracy and low cost. EIS can be measured in operational conditions [311]. Table 10 shows the different applications of EIS for LIBs.

Numerous innovative techniques have recently been developed and employed to investigate various battery components. Each method provides unique statistical insights. The three main measurement techniques used to study LIBs are operando, in situ, and ex situ [320]. “In operando” refers to experiments conducted under the actual operating conditions of a battery, simulating real-world usage. This method involves studying the battery while it is in operation, allowing for the observation of dynamic changes and behaviors during charge, discharge, and cycling without interruption. By providing insights into the battery’s behavior and performance under realistic conditions, this approach enables researchers to understand how various factors dynamically affect its operation [321,322]. Raman spectroscopy is a powerful tool for analyzing electrode structures in operando [323]. “In situ” refers to experiments conducted within the battery cell enclosure but outside operating conditions. This method allows for monitoring structural, chemical, or electrochemical changes within the battery without altering its environment [324]. In situ techniques can demonstrate the correlation between a battery’s microstructural and chemical evolution and its cycling parameters. These techniques allow researchers to observe and analyze the changes occurring within the battery’s internal structure and chemistry in real time as it undergoes charge and discharge cycles. “Ex situ” experiments, on the other hand, involve analyzing the components or materials of the battery outside of their original context, often after the battery has been disassembled [324]. In addition, many in situ and in operando methods have been introduced to characterize LIBs. These include neutron-related techniques [325,326], X-ray diffraction (XRD) [327,328], nuclear magnetic resonance (NMR) [329,330], and transmission electron microscopy (TEM) [331,332]. Other techniques such as scanning electron microscopy (SEM) [333], Raman spectroscopy, X-ray photoelectron spectroscopy (XPS) [334,335], and scanning transmission electron microscopy (STEM) [324] are also commonly used. In situ XRD is an appropriate analyzing method to characterize the structural changes in electrode materials during charge and discharge cycles that can provide information about phase transitions, crystalline structure, and phase composition. In situ and in operando, EIS can also be used to analyze the battery during operation. This technique allows for real-time monitoring of impedance changes, providing further insights into electrode/electrolyte interfaces, charge transfer kinetics, and ion transport phenomena during cycling. In situ and in operando X-ray absorption spectroscopy (XAS), including techniques such as X-ray absorption near-edge structure (XANES) and extended X-ray absorption fine structure (EXAFS), can also be employed to gather valuable data about the chemical state and local environment of elements in electrode materials during battery operation. This approach facilitates the understanding of redox reactions and phase transitions. Optical methods, including Raman spectroscopy and UV–Vis’s spectroscopy, have also found their position in the real-time monitoring of chemical and structural changes in electrode materials during battery cycling by providing valuable data regarding phase transformations, chemical reactions, and degradation mechanisms. SEM combined with techniques like Energy-Dispersive X-ray Spectroscopy (EDS) has shown great potential in the literature. NMR methods can also be utilized to analyze the dynamics and electrolyte decomposition processes of LIBs during operation, providing insights into ion transport and electrolyte stability. Furthermore, these techniques can be categorized as destructive (SEM, TEM, etc.) and non-destructive (in situ XRD, in situ EIS, in operando calorimetry, etc.) [336,337,338,339,340]. Also, the reliability of electrochemical techniques like cyclic voltammetry CV, EIS, and in situ/operando methods depends heavily on the quality of equipment, experimental setup, and data analysis. While CV offers quick qualitative results, it is less precise and sensitive than EIS, which provides highly reliable data for detailed kinetic and interfacial studies, though interpretation requires expertise. In situ/operando techniques offer the most realistic insights but are the most complex, demanding careful handling to ensure reliable results. Proper calibration and control experiments are essential for all techniques to obtain trustworthy data. Also, non-destructive techniques like in situ XRD, EIS, and XAS tend to offer better reliability for real-time monitoring and analysis of LIBs during operation. Destructive techniques like SEM and TEM, while providing valuable data, may limit continuous usage due to their invasive nature. The choice of technique ultimately depends on the specific information required and the acceptable trade-off between destructiveness and the depth of analysis.

Electrochemical characterization techniques for LIBs are crucial for understanding their performance and lifespan [341,342], whether in small-scale devices or larger systems like EVs and battery energy storage systems (BESSs). While the fundamental electrochemical principles remain the same, the challenges associated with thermal management, SOC estimation, and cell balancing become more pronounced. Characterizing these larger systems requires techniques that account for the heterogeneity in cell performance across a large number of cells connected in series and parallel. For example, advanced diagnostic tools are needed to monitor the individual cell health and identify potential anomalies early on. Non-destructive techniques such as ultrasound and infrared thermography can be particularly helpful for assessing the thermal profile of battery packs and detecting potential hotspots [343]. Finally, the development of comprehensive models is crucial for predicting the performance and lifespan of LIBs in larger systems. Electrochemical characterization data form the foundation of these models, allowing for the simulation of different operating conditions and scenarios. These models, which could encompass multi-physics aspects such as electrochemical, thermal, and mechanical behavior, are invaluable for designing robust and efficient EVs and BESSs.

By integrating advanced characterization techniques with sophisticated modeling, we can optimize battery system performance and reliability, extending the operational life of these critical energy storage technologies [343,344].

## 5. Challenges and Limitations

### 5.1. Issues Related to Cost, Resource Availability, and Scalability

**Figure 9:**
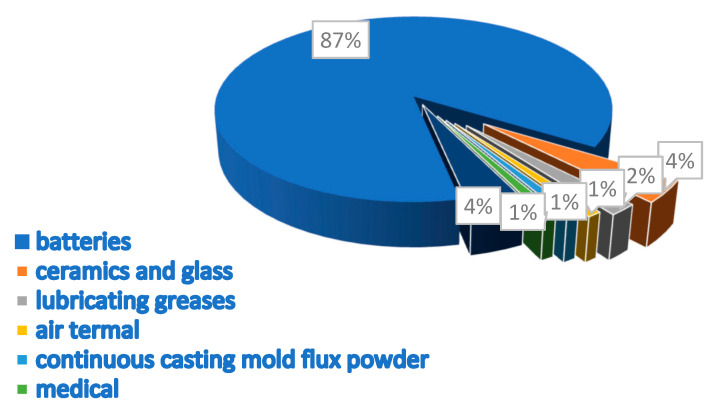
Key areas where lithium is utilized (2024) [349].

**Figure 10:**
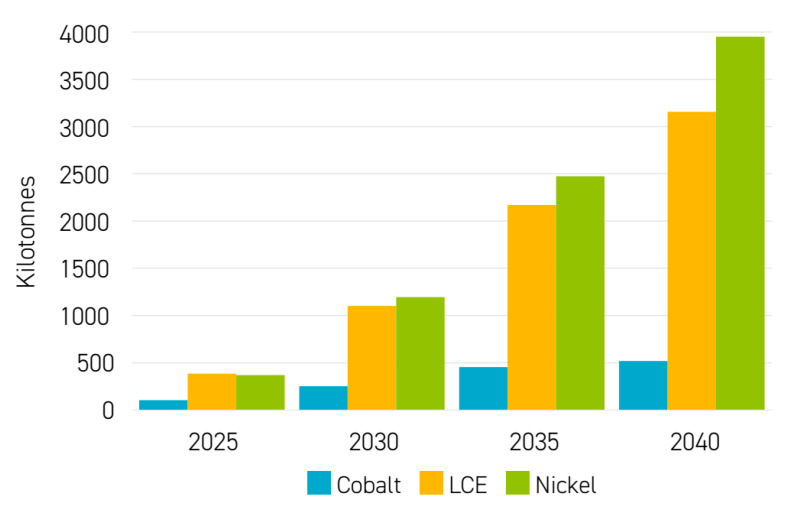
Global demand for raw minerals to 2040 including cobalt, lithium carbonate equivalent (LCE), nickel [351].

**Figure 11:**
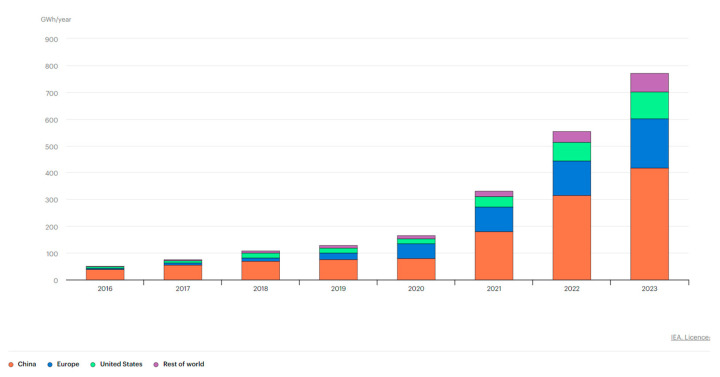
Stacked bar chart showing the EV battery demand from 2016 to 2023, broken down by region (China, Europe, United States, and rest of the world) [352].

The ban on petrol and diesel vehicles, coupled with achieving climate neutrality in Europe by 2050, necessitates transition in mobility. Several countries set specific targets for phasing out the sale of diesel and petrol cars: Norway aims for 2025, Ireland for 2030, and the United Kingdom and France for 2040 [345]. To make EVs more appealing regarding environmental, social, and health benefits, it is essential to consider factors like the criticality of raw materials and the competitiveness of European industries in the battery value chain [346]. China, Japan, and South Korea collectively account for more than 85% of the global supply of processed materials and components for LIBs. China in particular leads in Li-Ion cell manufacturing, producing approximately 75% of the world’s total cell output. In 2021, most lithium production was concentrated in China, Australia, and Chile [347]. The United States Geological Survey (USGS) provides valuable data on lithium production. Lithium is extensively used in various industries, primarily in batteries and glass/ceramic applications. Other significant uses include lubricants, continuous casting, air treatment, polymer production, aluminum production, and various industrial applications (Figure 9) [348]. Lithium can be sourced from multiple types of deposits, including lithium brines, sediment-hosted deposits such as hectorite and jadarite, pegmatites, and highly differentiated granite [349]. The global lithium supply chain is currently dominated by a few key players, each facing unique challenges. China, leveraging its domestic resources and longstanding supply contracts, maintains a strong foothold in the market. However, a significant concern arises from the anticipated decrease in lithium availability on the Chinese spot market due to the country’s surging domestic demand, mainly driven by the rapid growth of the EV industry. Simultaneously, South American nations like Argentina, Bolivia, and Chile, collectively known as the “lithium triangle”, possess substantial lithium reserves and remain a pivotal global market focus. Nonetheless, political instability and environmental disputes in these regions have posed obstacles to consistent and sustainable lithium extraction, further compounding the supply chain complexities. As the transition towards sustainable energy solutions accelerates worldwide, addressing these challenges and fostering a more diversified and resilient global lithium supply chain becomes imperative to meet the world’s burgeoning energy storage needs [348]. It is projected that by 2030, the European Union (EU) will require 18 times more lithium and 5 times more cobalt for EV batteries and energy storage alone. By 2050, these demands are expected to increase threefold compared to the current supply to the entire EU economy [350]. Figure 10 illustrates the substantial rise in demand for cobalt, lithium, and nickel. The graph highlights the attractiveness of adopting LIBs with cathode chemistries that feature very low or zero cobalt content, aiming to sustainably fulfill escalating energy storage needs. In 2023, global demand for EV batteries surged, driven by increasing EV sales, particularly in the United States and Europe, which saw growth rates of over 40%. The demand reached over 750 GWh, with electric cars accounting for 95% of this increase. Despite the growth, the U.S. remains smaller compared to Europe and China in terms of market size. The rising demand for batteries has intensified the need for critical raw materials like lithium, cobalt, and nickel. China continues to lead in battery production and exports, contributing to overcapacity issues but maintaining its dominance. Meanwhile, battery production is expanding in Europe and the U.S., with significant contributions from foreign companies. Figure 11 illustrates the regional demand for EV batteries from 2016 to 2023. As battery demand increases, there is a corresponding need for more extraction and refining of essential raw materials, particularly lithium, cobalt, and nickel.

**Figure 12:**
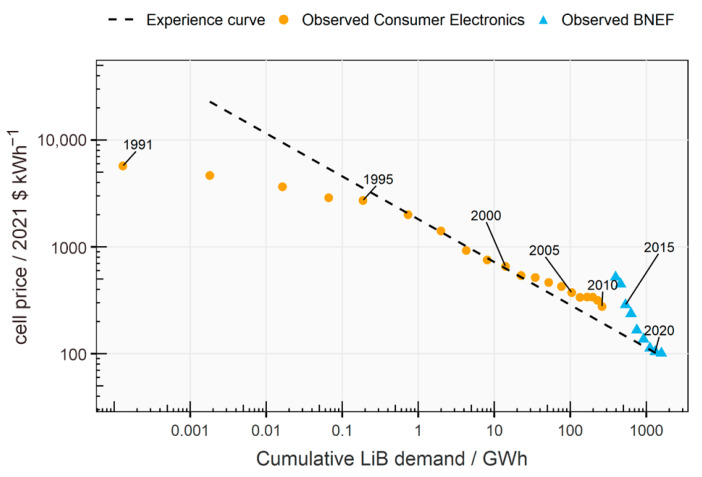
The prices of LIB cells from 1991 to 2021 [367].

The demands related to LIB in different applications, especially the transportation sector, motivated scholars to evaluate whether future raw materials supply related to LIB production can satisfy the material requirements. LIBs are expected to experience a significant surge in demand in the coming years. Ensuring an adequate supply of the critical metallic components employed in LIB manufacturing is crucial to meet this escalating requirement. Among these essential materials are manganese (Mn), nickel (Ni), and natural graphite (C). These metals play a vital role in the composition and functionality of LIBs. Consequently, their availability in sufficient quantities is imperative to sustain the projected growth and production levels necessary to satisfy the burgeoning demand for LIBs across various applications and industries [353]. Numerous studies have explored the future supply dynamics of raw materials essential for LIB production. Xu et al. [354] analyzed diverse market scenarios and raw material availability for the automotive sector, projecting their analysis until 2050. Similarly, Fu et al. [30] examined the supply prospects of raw materials employed in LIBs. While lithium, cobalt, and nickel are indispensable for optimal LIB functionality, these resources are relatively scarce [355], underscoring the importance of sustainable resource management and technological advancements to mitigate potential supply constraints in the burgeoning LIB industry. At the current consumption rate, the easily accessible lithium may be depleted shortly, increasing its price. This scenario also applies to nickel and cobalt, which are even more scarce than lithium [356]. Numerous countries, including Germany, Italy, China, India, France, Spain, Norway, South Korea, Japan, Sweden, the United States, and the United Kingdom, have introduced policies aimed at promoting the sales of EVs. These initiatives are expected to significantly increase the market share of LIBs in the rechargeable battery sector [349]. As the cumulative installed capacity of batteries grows, the production costs per kilowatt hour (kWh) are projected to decrease according to a power law driven by improvements in design, manufacturing techniques, and economies of scale. Nevertheless, the overall prices of batteries are determined by both material and manufacturing expenses. The reliance on costly materials such as cobalt, nickel, and lithium, vital for current battery technologies, will ultimately limit further reductions in production costs, setting practical lower boundaries for battery prices. Notably, cobalt prices have increased significantly in recent years [357]. The manufacturing of LIBs incorporates diverse materials beyond those already mentioned. From packaging to active substances, LIBs are composed of various metals and plastics, all of which influence these batteries’ overall cost and environmental impact [358]. According to Schmidt et al. [359], future prices of different electrical energy storage technologies can be anticipated through experience curves. Nykvist and Nilsson [360] discovered that the cost of LIBs in EVs decreased by around 14%. Various studies have utilized learning curves to model the trajectory of battery cost reduction. Learning curves are favored for simplicity, relying on only two variables: cumulative production volumes and historical prices. These findings highlight the importance of continuous cost reduction in battery production to make EVs more affordable and accessible to consumers [361]. The research also examines various battery chemistries’ technological and economic characteristics that are suitable for EV applications. It suggests a new concept where the cost of battery packs should follow a two-stage learning curve that eventually reaches a minimum price influenced by the expenses related to active materials [357]. LIBs have different properties in size, shape, voltage, and employed reactions. Their market has experienced rapid evolution, valued at USD 34.2 billion in 2020 and projected to reach USD 182.53 billion by 2030 due to the dramatically expanding EV market [353]. LIBs have undergone remarkable transformations driven by recent advancements in material science and electrode engineering. These breakthroughs have culminated in a substantial reduction in production costs, with per-unit expenses decreasing by an impressive 90%. Concurrently, the gravimetric energy density of LIBs has witnessed a significant uplift, surging from approximately 90 Wh/kg to an elevated level of 250 Wh/kg. This synergistic effect of cost minimization and enhanced energy density has positioned LIBs as a highly competitive and attractive solution for various energy storage applications [362]. The global production of lithium-based solid-state batteries is heavily concentrated in China, which has emerged as the world’s leading producer of LIBs. This dominant position can be attributed to China’s robust manufacturing infrastructure and strong governmental support for the EV industry. Prominent Chinese companies like CATL and BYD have propelled the nation to the forefront of battery manufacturing. Closely following China is South Korea, which commands a significant portion of the global lithium battery market. Major South Korean players such as LG Chem, Samsung SDI, and SK Innovation have played pivotal roles in producing and advancing LIB technology worldwide [45]. In addition, Japan has maintained a notable presence in Li battery production, leveraging its technological expertise and automotive industry prowess. Countries such as the United States, Germany, and Taiwan contribute to LIBs production, but their shares are comparatively smaller. Several studies have forecasted the future demand for lithium, an essential element in LIBs. Ziemann and Müller [363] estimated that the lithium demand would reach 600,000 tons annually by 2050. Mohr, Mudd, and Giurco [364], focusing specifically on EV batteries, projected a lithium demand of 400,000 tons annually by 2050. In another study, Maxwell and Mora [365] predicted a lithium demand of around 500,000 tons per year by 2025, which further escalates to 1,500,000 tons annually by 2037. Lithium has emerged as the most critical limiting element in LIB batteries, exemplified by its scarcity in LiFePO_4_ chemistry, where it serves as the limiting component. The calculation assumes a battery with a capacity of 40 kWh and considers the complete utilization of the market for the critical element involved in battery production. Most battery chemistries appear capable of meeting short-term (10–15 years) availability goals, but in the case of long-term (40–50 years) EV targets, significant production expansion should be taken into consideration, as stated by Egbue, Long, and Kim [366]. Figure 12 illustrates the cell prices of LIBs from 1991 to 2021. As depicted in this figure, the price of LIBs has exhibited a relatively downward trend over this period. This consistent decline in cost can be attributed to advancements in material science, electrode engineering, and increased manufacturing efficiency. The trend underscores LIB production’s progress and economies of scale, making them more economically viable for various applications, particularly in the EV industry.

### 5.2. Safety Concerns: Thermal Runaway, Dendrite Formation, and Mitigation Strategies

**Figure 13:**
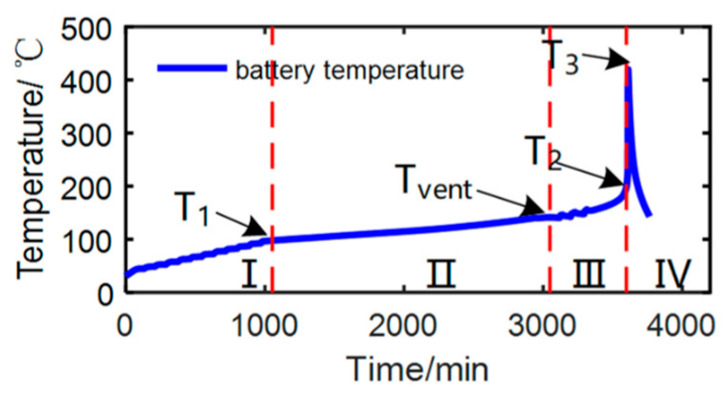
The stages of thermal runaway: I: Initial Heating, II: Exothermic Reaction Initiation, III: Thermal Acceleration, IV: Runaway [371].

Although LIBs offer numerous benefits, they also present certain drawbacks that must be addressed. One primary concern is their optimal operating temperature range, ideally between 15 °C and 35 °C. Operating these batteries outside this range can lead to short circuits, possibly causing a hazardous and potentially catastrophic thermal runaway [368]. Therefore, much scientific research on LIB safety is dedicated to understanding and preventing thermal runaway scenarios [369]. To ensure the safety of EVs, many countries enforce robust regulations requiring manufacturers to conduct rigorous safety testing. This includes evaluating batteries for their ability to withstand various environmental stresses such as impacts, extreme temperatures, and vibrations. As manufacturers push LIBs to their limits for higher electrochemical power, new abuse conditions have emerged, specifically termed electrochemical abuse. Thermal runaway is a phenomenon often triggered by mechanical, electrical, or thermal abuses, each of which poses significant safety hazards [370]. Mechanical overuses consist of collisions, crushes, and nail penetrations, and electrical overuses are overcharging, over-discharging, and external short circuits. Thermal overuse causes overheating, high ambient temperatures, or fires to occur. This overuse can cause separator collapse followed by internal short circuits (ISCs). The ISCs may lead to overheating of the battery, intensifying electrochemical side reactions, and finally releasing flammable gas, raising internal pressure and the expansion of the battery, potentially leading to fire. The central flammable part of LIBs is their liquid electrolytes, which cause more explosion risk. Thermal runaway of LIBs can occur in three steps of temperature rising: T1, the onset of battery self-heating; T2, the trigger temperature of thermal runaway; and T3, the maximum temperature. T1 can occur due to SEI decomposition, triggered when the battery’s temperature surpasses a threshold. However, T2 is known as a separator failure, resulting in ISC, marked by a shift from a constant to an exponential temperature rise. Finally, T3, the highest temperature point, has been considered the peak of thermal runaway. Many models can be used to simulate different segments of the temperature profile, from T2 to T3 or the entire profile from T1 to T3. Figure 13 illustrates the battery temperature progression and the various stages leading to thermal runaway.

Ren and colleagues [372] evaluated the relationship between ISCs and thermal runaway in LIBs under thermal overuse conditions based on experimental and modeling approaches. They demonstrated that the ISCs commonly occur before thermal runaway or contribute to heat generation minimally. Moreover, thermal shrinkage of the separator in the battery was also shown to be the reason for ISCs. The exothermic reactions between the anode and electrolyte trigger thermal runaway, which has been detected as having a negative value contrary to conventional beliefs. In another study, Jia et al. [373] created a model for thermal runaway propagation by conducting numerous experiments and mathematical modeling tasks. They posited that critical factors like ambient temperature, packing spacing, and stacking arrangement can impact the propagation speed. Researchers have indeed pointed out a detailed correlation between engineering design variables and the thermal runaway behaviors of a particular battery pack. Currently, thermal shutdown separators are a common strategy for LIB safety; however, conventional designs using low-melting-point polymers like polyethylene (PE) and polypropylene (PP) are often insufficient to prevent thermal runaway. In contrast, thermal obturator separators, which undergo phase transitions at elevated temperatures, offer an alternative mechanism. These separators are designed to seal the pores within the membrane upon reaching a critical temperature, effectively interrupting ion transport and thereby mitigating temperature escalation [374]. Chen et al. [375] proposed using an optimal thermocouple to enhance the accuracy of battery thermal runaway performance evaluation. A significant challenge in commercializing LIBs with lithium metal anodes is the formation and unpredictable growth of lithium metal dendrites [376]. Machine learning methods can improve safety management for LIBs in EVs by analyzing real-time data to forecast operational conditions and detect potential hazards, such as thermal runaway [377]. During the charging process, lithium ions are irregularly deposited on the anode surface, forming these dendritic structures. The presence of dendrites not only deteriorates battery performance by disrupting uniform current distribution but also raises severe safety concerns. Dendrites can penetrate the separator between the anode and cathode, potentially causing ISCs and triggering thermal runaway, fires, or explosions. Moreover, the lithium consumed in dendrite growth reduces the available capacity for electrochemical reactions, resulting in low cycling efficiency over repeated charge–discharge cycles [378,379,380]. To prevent irreversible damage and safety risks from LIB overcharging, using an electroactive polymer separator is a promising method due to its ability to reversibly switch between conductive and insulating states under high overcharge conditions [381]. Fast charging in an inappropriate temperature range causes the growth of dendrites, which causes ISC. Researchers have focused on packing more energy into the same volume for LIBs to increase energy density while maintaining cost effectiveness. However, this energy manipulation inevitably leads to inherent safety issues [382]. Consequently, extensive research has been conducted to understand the fundamental causes of dendrite formation and develop methods to prevent its growth. Recent advancements in this area include the utilization of ionic liquid electrolytes [383], MOF-modified electrolytes [384], and electrolyte additives [385] to control dendritic growth. Additionally, exploring stable lithium hosts [386] and engineering the interface on lithium metal [387] has shown promising results in mitigating this challenge. Thus, considerable research has focused on understanding the fundamental causes of dendrite formation and developing methods to prevent their growth. By comprehending these failure mechanisms, we can devise effective mitigation strategies. Various approaches are employed to ensure the safety of LIBs, addressing specific failure mechanisms and enhancing overall safety. Ensuring the safety of LIBs remains a paramount priority, and numerous measures are implemented to maintain their integrity and performance. Improving the stability of LIBs can be achieved by changing the battery’s chemistry or component structures. Limiting the current flow during operation is also a safety measure. Adding specific safety features to the battery design is another way to ensure safety, with a focus on cost effectiveness and avoiding packaging issues. Managing the thermal runaway is crucial, as delaying this event in case of battery failure offers individuals more time to escape potential harm. Properly managing the battery’s temperature is essential to prevent dangerous heat buildup [388]. Advanced thermal management strategies are crucial for enhancing the efficiency and longevity of electronic devices, particularly in high-performance applications such as data centers, EVs, and aerospace systems. These strategies encompass a variety of approaches, including passive cooling techniques, phase-change materials, etc., that optimize heat dissipation. Selecting the appropriate thermal management system for LIBs is crucial, taking into account elements like battery rates of charging and discharging, lifespan, and dimensions.

Maintaining optimal operating temperatures (15–35 °C) in LIBs is crucial for safety and longevity, as high temperatures and inconsistencies can lead to accelerated aging and thermal runaway. Various cooling systems exist, including air-based [389], liquid-based [390], phase-change material (PCM)-based [391], and heat pipe-based [392], each with advantages and disadvantages. Air cooling is simple but may require forced convection; liquid cooling offers better heat transfer but direct immersion is not typically used in EVs due to safety concerns. Factors like coolant type, cell arrangement, and flow paths significantly impact cooling efficiency, and optimizing parameters such as inter-cell spacing and flow rate are essential for effective and uniform temperature management. The most commonly used approach for thermal management in EVs is air cooling systems for BMSs. These systems are favored due to their easy installation, low production costs, and minimal maintenance requirements. Liu et al. [393] introduced a thermoregulating separator for LIBs that incorporates a PCM to absorb heat during abuse conditions. Ping et al. [394] recommended enhancing the thermal management structure of a battery pack by adding fins to the outer surface of the PCM to boost its thermal conductivity. A hybrid cooling approach, as proposed by Yao et al. [395], combines liquid cooling channels with PCMs to achieve both high thermal efficiency and low-pressure loss. Despite advancements in the development of these materials that enhance the safety of LIBs, they have not effectively resolved the fundamental issue of thermal runaway or achieved intrinsic safety. Also, cooling systems prove insufficient. Early thermal runaway warning systems are crucial for timely intervention and preventing propagation, as demonstrated by studies showing significant time gains in mitigating thermal runaway events. Monitoring electrical characteristics, temperature, gas, and force changes are vital for effective early warning mechanisms [396]. The conventional BMS has limitations in monitoring internal battery conditions, failing to detect internal chemical changes despite abnormal external characteristics. Advanced techniques like X-ray diffraction and nuclear magnetic resonance are effective but require specialized equipment, making them unsuitable for commercial cells. To address this limitation, recent innovations have integrated optical fiber sensing techniques at the cell level, providing valuable insights into internal parameters. Su et al. [397] have developed a real-time, non-destructive battery safety system equipped with a potential sensing separator, which effectively monitors anode liquid phase potential and detects early battery defects, ensuring thermal safety and operational stability over prolonged periods. This novel approach minimizes structural impact and presents a promising solution for next-generation BMS. Zhang et al. [398] conducted a study that emphasizes the importance of thermal management systems for LIBs, particularly in applications like EVs and ESSs. The research highlights the necessity of battery thermal management systems (BTMSs) to maintain LIBs within an optimal temperature range. The study underscores the role of BTMSs in ensuring the sustainable and safe development of LIBs, which is critical for their effective use in high-demand applications. A review by Lin et al. [399] aims to offer a thorough understanding of the fundamentals of BTMS. It addresses the challenges related to temperature sensitivity in LIBs and highlights the advancements in two-phase heat transfer technologies for BTMS. While LIB thermal management has improved, significant obstacles still require further investigation. The field of battery efficiency and safety is constantly evolving, driven by the rising demand for enhanced capabilities and longer lifespans, especially in EV applications. Table 11 summarizes various mitigation strategies that can be adopted to enhance the safety of LIBs.

### 5.3. Environmental Impact and Sustainability Challenges

**Figure 14:**
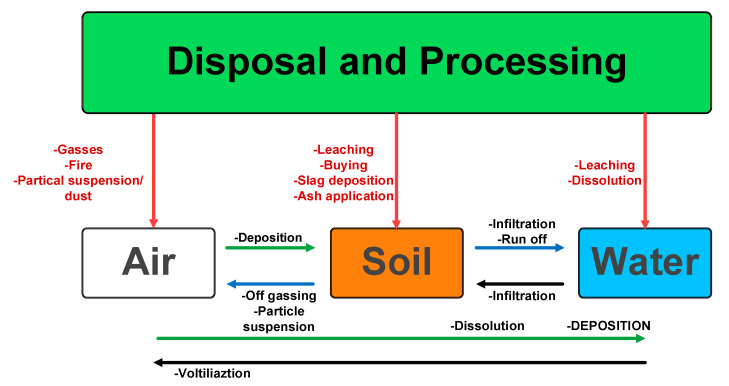
The emissions from the LIB industry to the environment [445].

The global population, now reaching 8 billion, is driving a significant increase in energy demand, particularly for rechargeable batteries used in EVs and portable electronics. As a result, there is a surging demand for these batteries across various applications [425]. Their growing integration into everyday life has heightened awareness of potential environmental issues stemming from their manufacture and disposal [426]. The issue has been extensively examined by scholars, resulting in a substantial body of research exploring the potential environmental impacts and sustainability of lIB [427,428,429,430]. The economic and environmental challenges arising from the current utilization of lithium batteries are closely interconnected [431]. The production of LIBs inevitably leads to an increase in the number of spent (or used) batteries because these batteries have a finite lifespan, typically ranging from 3 to 10 years. This means that after this period, these batteries can no longer efficiently hold a charge or perform their intended function and thus must be replaced or recycled. As the production and usage of LIBs continue to rise, so does the quantity of these spent batteries that require proper disposal or recycling to manage environmental impact and resource recovery [432]. The sustainability challenges inherent in the LIB value chain are multifaceted and warrant comprehensive examination [433]. Studies on LIB sustainability have five key limitations: lack of comprehensive analysis, reliance on pre-defined frameworks, failure to prioritize sustainability aspects, lack of stakeholder involvement, and neglect of feasibility in practice. These limitations include neglecting essential aspects like circularity and assuming equal importance of different sustainability issues. The studies also often overlook the dynamics of stakeholder interactions, governance structures, and data availability, which are crucial for implementing meaningful supply chain-wide assessments [434]. Supply chain complexities (SCCs) represent another significant challenge in the LIB industry [435]. The geographically dispersed nature of critical raw materials, coupled with intricate global supply networks, renders the industry vulnerable to disruptions. Geopolitical factors, political instability in key resource-producing regions, and transportation logistics all play critical roles in determining the reliability and cost of supply chains. Moreover, the increasing demand for LIBs necessitates robust and resilient supply chain management strategies to ensure adequate material availability and on-time delivery. These characteristics lead to intricate cause–effect relationships that complicate navigation and decision-making within organizations. The interplay of these complex elements can yield various hidden effects, influencing aspects such as capacity utilization, demand fulfillment, inventory levels, and resilience. Ignoring these supply chain dynamics undermines realistic projections of battery availability and deployment scenarios. The Ren et al. [436] study focuses on the LIB industry, reviewing evaluation methodologies and proposing a comprehensive approach. Their findings highlight that production and usage stages have the greatest environmental impact within the industry. Based on this, they suggest an evaluation system that considers environmental impact, resource criticality, economic analysis, and material flow. This framework aims to guide the industry towards sustainable practices and a low carbon footprint while also noting the potential profitability of recycling waste batteries. Bokrantz et al. [437] research investigates the challenges of SCC within the maintenance operations of Europe’s emerging LIB production sector. The study highlights the significant impact of SCC on maintenance and provides guidance for managing it. Ultimately, this research positions SCC as a valuable theoretical framework for understanding the complexities of the growing battery industry. Primarily, the extraction and mining of critical raw materials, such as lithium, graphite, and cobalt, raise significant environmental and ethical concerns, particularly regarding cobalt sourcing. In contrast, the sustainability issues associated with battery cell manufacturing processes have not been as extensively explored in existing research. Some materials used in battery manufacturing, classified as critical raw materials by the EU, pose environmental concerns despite lacking heavy metals like lead or cadmium and often fall short of sustainability and green chemistry standards [429]. The expansion in lithium material production depletes natural resources and leads to environmental issues such as pollution from mining and mineral processing activities, ecosystem degradation, and greenhouse gas emissions [431]. Lithium extraction has considerable environmental repercussions, notably a high risk of water contamination affecting 65% of lithium mines. In response, several countries have strengthened existing regulations or introduced new ones, mandating mining companies to disclose their environmental, social, and governance (ESG) performance in sustainability reports [438]. Transitioning away from fossil-fueled vehicles in favor of EVs is seen as a crucial strategy for combating climate change. As a result, numerous governments around the world are embracing this shift toward greener transportation options [439]. In response to concerns about monopolization and the unstable supply of battery materials, governments globally are adopting various strategic policies tailored to their roles in the battery value chain. These measures encompass enhancing domestic production, diversifying suppliers, bolstering refining and recycling capabilities, fostering technological advancements, and promoting responsible sourcing to mitigate environmental and social risks [439]. Sankar et al. [428] analyze the environmental impacts of LIBs, from raw material extraction to disposal and recycling. They emphasize that reusing recovered materials in battery production can significantly diminish environmental footprints, greenhouse gas emissions, and energy use, highlighting the importance of improving recycling and disposal practices, including ecosystem destruction during mining and the environmentally damaging processes involved in metal extraction and LIB manufacturing [440]. It is crucial to acknowledge the significant environmental impact of energy sources, as gasoline combustion, for instance, releases substantial amounts of CO_2_ [431]. Disposing of used batteries can lead to serious environmental hazards, with the potential for widespread ecological damage. It is crucial to manage these batteries effectively, which calls for practical strategies. One of the most effective solutions is to develop strong recycling programs for batteries [425,441]. Spent LIBs also show valuable metals like lithium, copper, nickel, aluminum, and cobalt, making recycling essential for reducing pollution and supplementing metal sources. It is estimated that 200–500 million tons of spent LIB wastes was produced annually worldwide in 2022, and landfilling these spent batteries can be associated with significant environmental and health risks due to their flammable organic and toxic substances [432]. The cathode material makes up roughly 50% of LIB costs due to high-value metal elements. Recovering these metals from spent cathodes is essential for ensuring the economic feasibility of recycling processes [442]. Materials recovered from spent LIBs can be reused in diverse applications, such as the creation of new batteries and electronic components [443]. Critical metals, including cobalt, nickel, and lithium, can be reprocessed for battery production, while graphite may be repurposed for lubricants and carbon materials. Although promising alternatives exist for using these secondary materials, a noticeable gap persists between research advancements and their practical application in industry [36]. The EU is implementing new battery regulations that mandate all batteries to come with a footprint report before being allowed into the market by July 2024. Additionally, by 2026, it will be compulsory for batteries to carry energy consumption labels. In summary, the manufacturing and disposal of batteries have serious environmental consequences, prompting regulatory measures to address these issues [444]. As stated by Mrozik et al. [445], many detected hazardous pollutants are released from LIBs, including vapors, gases, metal nano-oxides, and electrolyte degradation products that can bring out many threats to soil, water, and air. Battery production has been found to have substantial effects on global warming, carcinogens, ozone depletion, and ecotoxicity. Additionally, the recycling of batteries has a considerable environmental and resource impact [446]. Hence, many efforts have been made to address the knowledge gaps to mitigate the negative environmental impact of LIBs, and replacing toxic metals with less toxic ones in battery materials may enhance environmental sustainability. Researchers have extensively studied the environmental consequences of LIB production and electric mobility using LIBs, with numerous available studies. To assess the potential environmental advantages, these studies employ life-cycle assessment (LCA), a standardized method for evaluating the environmental impacts of products or processes across their entire life cycle [447,448]. In a comprehensive LCA study, various environmental impact categories are considered, including global warming potential, ozone depletion potential, terrestrial acidification, fossil depletion potential, potential particulate matter formation potential, photo-oxidation formation potential, freshwater toxicity, marine eutrophication potential, and freshwater eutrophication potential [432]. Looking ahead, the battery industry is anticipated to focus on reducing the environmental footprint of used batteries by developing components that are biodegradable or environmentally benign [449]. However, as mentioned in [450], it is crucial to devise new, straightforward, cost-effective methods with higher recovery rates for valuable metals from spent LIBs to secure a secondary supply. In this context, aqueous rechargeable batteries emerge as an up-and-coming solution [451]. Existing literature lacks adequate quantification of the impacts necessary for critically assessing and comparing various recycling methods. Currently, researchers classify the waste materials from LIBs as hazardous, especially those from EVs, because of the possible risks to environmental and human health due to releasing heavy metals. The waste LIBs, due to their chemical structure (many chemicals such as volatile organic electrolytes, reactive salts, and additives), showed many dramatic effects on the environment. In addition, the side pollution from the battery combustion process can lead to many gaseous air pollutants or sometimes leach into the soil, groundwater, and surface water [452]. Current battery technologies, relying on finite resources materials, face critical challenges related to environmental impact and safety. Dühnen et al. [453] propose two strategies aimed at enhancing the sustainability of electrochemical energy storage systems. For applications such as EVs, the primary focus is on improving the sustainability of LIBs. This can be achieved through extending battery lifespans, facilitating second-life applications, and implementing efficient recycling processes, in addition to making incremental improvements in manufacturing techniques. There are significant uncertainties regarding LIB growth predictions, and it is argued that strong governance and effective regulations at both national and international levels are essential to ensure the long-term sustainability of LIB materials. LIB materials can be stored in a “metal bank” to protect against supply shocks and fluctuating costs. Oil stockpiling has been used as a strategy to stabilize prices during volatile periods, and a similar approach could be applied to LIB materials. Figure 14 illustrates the probable paths through which pollution spreads from the disposal or processing of end-of-life LIBs into the environment, highlighting the potential for cross-contamination between various compartments.

### 5.4. Regulatory and Standardization Hurdles in LIB Manufacturing and Recycling

Lack of regulations leads to most end-of-life batteries in landfills, but proactive policies and recycling advancements could make them a significant source for new battery materials. The EU Batteries Regulation, effective 2024, mandates a specific percentage of recycled materials in new batteries starting in 2031, with goals to increase Europe’s battery recycling capacity from under 4 GWh (projected for 2025) to 200 GWh by 2040. By 2031, battery manufacturers must incorporate 6% nickel, 16% cobalt, 85% lead, and 6% lithium from recycled sources, with these figures rising to 15%, 26%, 85%, and 12%, respectively, by 2036. To facilitate informed consumer choices, a labeling system incorporating QR codes will link to digital passports offering detailed battery information. Companies are required to mitigate social and environmental risks associated with sourcing raw materials such as lithium, cobalt, and nickel. With an anticipated surge in battery demand within the EU, it is crucial that these needs do not exacerbate existing environmental challenges. Up to 54% of components in LIBs are recyclable, yet material sourcing remains concentrated in a few countries, complicating procurement. This regulation is a significant element of the European Green Deal, supporting the EU’s objectives for a circular economy and pollution reduction. It seeks to ensure sustainability throughout a battery’s lifecycle, enhancing the development of a competitive battery industry while fostering Europe’s clean energy transition and reducing reliance on fuel imports. However, experts caution that the ambitious recycling quotas may be difficult to achieve given the longevity of EV batteries and the rapidly growing demand for battery technologies [454]. In 2024, the USGS reported that around 87% of global lithium consumption was dedicated to battery applications, encompassing rechargeable LIBs and non-rechargeable lithium primary batteries [349]. In addition to focusing on component recycling approaches, there is an increasing spotlight on the reutilization of LIBs within the LIB circular economy [455]. Recognizing the environmental consequences during end-of-life stages and executing sustainable waste stream management practices are critical [456]. This review consolidates data about the qualities, ecological consequences, and managerial approaches concerning the end-of-life waste stream of LIBs [455]. Based on analysis by Richa et al., estimates of LIB waste generated from EVs can be made by considering parameters like EV sales figures, projected EV lifespans, and average LIB weights [457]. When the capacity of EV batteries drops below 80% of their rated level, they are considered for retirement [456], which could potentially find reuse in stationary ESSs such as grid-scale storage, EV charging stations, or telecommunications infrastructure [458]. Given the projected rise in the global EV count to 140 million by 2030, managing retired EV batteries efficiently is crucial for waste management and ensuring a sustainable resupply chain [459]. The obstacle with recycling LIBs is the low collection rate, often due to the lack of economic incentives [460]. Recycling is a post-consumer process focused on achieving material reintegration into circular economies. A novel approach proposes incorporating product design principles into recycling methodologies to optimize efficiency [460]. This broader perspective, relevant to various complex products, underscores the significance of design in enhancing recycling practices [459]. The recycling processes for LIBs have been thoroughly examined, revealing the substantial impact of battery design on recycling efficiency and costs. Before the recycling stage, it is crucial to safely discharge the batteries to mitigate the risks of short circuits and self-ignition during the disassembly process [449,461]. The general structure for the established recycling methods for LIBs is evolving [462]. Kim et al. specifically examined the recycling processes of LIBs [463]. In recycling LIBs, three primary methods can be utilized: direct recycling, pyrometallurgical methods, and hydrometallurgical methods [449]. In previous literature, the efficiency of mineral recovery through hydrometallurgical recycling has been notably higher than with other methods [464]. Various challenges hinder the development of a universally accepted battery recycling technology. These obstacles include the disparity in LIB sizes, the diverse nature of manufacturing processes, and limited battery components [465]. The practical considerations surrounding LIB production are often overlooked in theoretical discussions [466]. High production yields are crucial for economic viability, demanding rigorous quality control at each stage, from raw material sourcing to cell assembly and testing. Variations in material properties and manufacturing processes directly impact the final product’s performance characteristics, including energy density, lifespan, and safety. Consistent quality control, including advanced analytical techniques and automated inspection systems, is essential to minimize defects and ensure consistent performance across large-scale production runs. The absence of detailed analysis in these areas hinders accurate cost projections and informed decision-making.

Industrial sources indicate that the current recycling capacity is underutilized, highlighting the necessity to centralize battery supply to address this issue efficiently. The comprehensive evaluation criteria for battery recycling are centered on three main pillars: efficiency, economic viability, and environmental impact, with a crucial safety assessment component. Regulations are necessary to preserve our societies and the environment [467]. Numerous countries have dedicated considerable resources to advancing technologies for LIB recycling. A legal discussion proposes a framework for regulations concerning battery recycling [468]. Numerous safety regulations have been established to mitigate risks associated with their production and use to ensure the safe operation and storage of LIBs. These regulations mandate compliance with specific criteria and promote sustainable practices such as resource conservation, emission reduction, and material recycling. These regulations are critical to supply chain transparency and essential for ethically sourced raw materials like lithium, cobalt, and nickel. However, adhering to these regulations can add complexity to supply chain management, requiring detailed tracking and reporting of material sources and practices. In Europe, a forthcoming regulatory framework aims to comprehensively oversee the manufacturing, labeling, reuse, and recycling of LIBs to promote technological advancement. This proposal requires manufacturers to provide data on battery durability, performance, and material sourcing to ensure transparency [469]. While Europe sets broad guidelines, national policies vary: the UK currently lacks explicit LIB recycling regulations [470], whereas Germany has established a regulatory system for LIB recycling [465]. In Asian countries such as Japan and China, various approaches to LIB recycling are employed. China has introduced various regulations and policies to manage solid and hazardous wastes [471]. Conversely, Japan has established a comprehensive set of laws that govern battery recycling. These laws oblige new vehicle manufacturers to take responsibility for the batteries and stay updated on recycling technologies. In Japan, the Japan Portable Battery Recycling Center (JBRC) supervises battery recycling operations and advocates for reusing batteries for home emergency power [467]. Similarly, India has specific regulations in place for electronic waste management [472], while Australia currently lacks a LIB recycling policy [473]. Regulatory progress regarding battery recycling is limited in countries like the United States and India. However, the United States is growing interest in battery recycling at the federal level. Battery manufacturers globally are refining the standardized manufacturing processes and considering the economic and environmental consequences. Despite established policies in countries like China, Japan, the EU, and the US, there is still a lack of explicit systems and regulations for effectively recycling spent batteries [474,475]. To accelerate the expansion of LIB recycling, the government must prioritize funding for research and development (R&D), support pilot projects, and implement market-driven initiatives. These actions are essential for creating an attractive environment for investment in LIB collection and recycling [476].

## 6. Emerging Trends and Future Directions

### 6.1. Advanced Battery Technologies: Lithium-Sulfur (LSBs), Lithium-Air, and Alternatives to Lithium-Ion

Given the various drawbacks of LIBs, it is crucial to explore next-generation battery chemistries that can exceed the current standards in performance, energy density, and safety. Pursuing these advanced batteries should involve a rational strategy to develop sustainable long-term solutions. This includes utilizing abundant materials and eco-efficient production methods. Lowering costs facilitates broader market adoption, resulting in significant socio-economic advantages [451]. These advancements encompass solid-state, lithium-air, LSBs, and sodium-ion batteries. LSBs in particular are highly recommended due to their promising potential as “Beyond LIBs”, offering high-capacity sulfur chemistry with achievable capacities exceeding 500 Wh/kg. Solid-solid state batteries can be used to develop current EV batteries and other applications. The current LSBs have many challenges that should be concentrated on, including achieving high sulfur loading in the cathode and the issues related to the lifespans of these batteries. Dealing with challenges about LSBs, including sluggish polysulfide reaction kinetics and the polysulfide shuttle, has very high study potential. In addition, using different strategies like mediator redox conversion and stepwise catalyst concepts can also be very promising. One aspect that has received less attention in the literature is the development of large-scale processes, and most current systems are in their small-scale situation [477]. Additionally, further advancements at the material level are essential to harness the potential of lithium-sulfur (Li-S) technology fully. The academic research community plays a crucial role in this effort, and a significant portion of material-level research must focus on developing a viable Li-S cell system [478]. Cha and colleagues [479] suggested utilizing carbon-based nanomaterials to enhance sulfur conductivity and prevent polysulfide migration and volume change. However, these materials have notable drawbacks, such as adding significant weight to the cathode and causing sulfur detachment due to their non-polar surface. Therefore, research into polar and electrocatalytic materials to accelerate polysulfide conversion is fundamental and beneficial for maintaining stability. Effective electrocatalysts must prevent unwanted side reactions and allow for higher sulfur loading. Future studies should focus on exploring fundamental scientific principles, innovative technologies, and advanced materials at the cellular level to increase discharge capacity closer to theoretical limits, along with innovations to regulate chemical equilibria among active species at the nanoscale. Also, solid-state batteries (SSBs) using solid electrolytes (SEs) offer safety, high energy density, and low cost. Polymer electrolytes, like PEO-based materials, are promising but suffer from low ionic conductivity due to crystallinity. Challenges remain, including dendrite formation and potential Li_2_CO_3_ formation [429]. LIBs are the most used capacity technology in the EVs industry. The Blade battery is a revolutionary LIB that boasts a unique design, resembling a blade, and offers several advantages over traditional LIBs. Its innovative structure, comprising thin LFP sheets stacked together, enables a more compact and efficient battery with improved thermal stability [480,481]. This design allows for higher energy density, resulting in an extended driving range of up to 600 km on a single charge and a longer lifespan of up to 1.2 million kilometers. The Blade battery’s safety features are a significant improvement over traditional LIB. Its built-in thermal management system prevents overheating, and its unique design prevents thermal runaway, the leading cause of battery fires. Additionally, the battery’s electrolyte solution is less volatile, reducing the risk of fires. The Blade battery also features a robust battery management system that monitors its performance and temperature, shutting down the battery if abnormalities are detected. These safety features, combined with rigorous safety testing, make the Blade battery an attractive option for EV manufacturers seeking to improve their vehicles’ safety and reliability. The Blade battery’s safety advantages are further enhanced by its use of LFP, which is significantly safer than batteries based on NMC oxide. The BYD nail penetration test demonstrates the Blade battery’s high level of safety, with LFP cathodes featuring phosphate poly-anions that are resistant to breakdown, even at high temperatures. This results in a minimal amount of oxygen being emitted during nail penetration, reducing the risk of thermal runaway [482]. Overall, the Blade battery’s innovative design, high energy density, longer lifespan, and improved safety features make it a significant development in the field of EV batteries, with the potential to become a global standard for EV batteries.

### 6.2. Advanced Electrode and Electrolyte Materials for Enhanced Performance and Safety

Advanced electrode and electrolyte materials are crucial for enhancing the performance and safety of LIBs. These materials significantly improve various factors, such as thermal stability, energy density, and cycle life while reducing safety risks. Recent studies have focused on developing new electrode materials with higher capacity, enhanced rate capability, and better stability. Examples include silicon-based anodes and high-nickel cathodes [483,484]. Additionally, numerous studies have aimed to improve electrolyte formulations to boost ion conductivity, suppress dendrite formation, and enhance thermal stability. These advancements are vital to meet the increasing demand for high-energy-density and safe LIBs in applications like EVs, portable electronics, and grid ESSs [485,486,487]. Furthermore, aqueous zinc-ion batteries (ZIBs) are being explored as potential next-generation ESSs due to their high zinc content, safety, eco-friendliness, and high specific capacity. ZIBs offer benefits such as enhanced safety and lower costs. However, their practical applications are limited by the vulnerability of their cathodes in many scenarios. To solve this issue, Lee et al. [488] constructed and evaluated a new cathode comprising free-standing manganese oxide (MnO_2_) on a flexible graphene film for aqueous ZIBs. Their research demonstrated outstanding electrochemical performance, achieving a high energy density of 396 Whkg^−1^. Yoo et al. [489] demonstrated the application of electrospray technology to create a porous film on LIB cathodes, which led to improved electrochemical performance and cycle efficiency. Their study highlights the potential of ES technology in advancing energy storage solutions. The integration of electrospinning and electrospray deposition techniques represents a significant innovation in the development of porous cathodes for LIBs. Electrospinning employs high voltage to create nano- to micro-scale fibers from a polymer solution, resulting in a high surface area and a porous electrode structure. Critical to this process is maintaining low solution concentrations, which allows for fine control over electrode morphology and facilitates the creation of large-area porous films. In electrospray deposition, charged droplets with suspended materials are directed toward a substrate, ensuring uniform deposition with minimal waste. This approach significantly enhances ionic conductivity, as the increased porosity permits reactions throughout the electrode, promoting uniform degradation and improved cycle stability. Compared to traditional blade coating methods, electrospinning yields a more porous surface and better distribution of conductive agents, enhancing charge transport and overall electrochemical performance. Overall, this innovative method addresses key challenges in electrode design, potentially leading to advanced LIB performance characterized by higher efficiency and longer cycle life [489,490]. Maintaining the operational integrity of LIBs necessitates a significant emphasis on enhancing the thermal resilience of their electrolyte systems. Failure modes observed in these batteries underscore the critical role of electrolyte composition, specifically the inherent instability of organic carbonate solvents and certain lithium salts at elevated temperatures. Contemporary research efforts are strategically directed toward several key avenues: the synthesis of robust lithium salts characterized by superior thermal stability; the incorporation of functional additives designed to impart non-flammability to conventional electrolyte solvents; and the exploration of alternative, intrinsically non-flammable solvent chemistries [491]. Traditional liquid organic carbonate-based electrolytes in batteries are flammable and thermally unstable, posing safety hazards. Improving their flammability point or adding fire-retardant additives can mitigate these risks. Radical scavengers can also reduce flammability by interrupting combustion reactions. Solid-state electrolytes offer a safer alternative due to their superior stability [492,493]. Cathode interface stability can be improved by techniques like element doping and the addition of film-forming or high-voltage electrolyte additives [213,494]. Several modified separators have been created to enhance safety, categorized into inorganic ceramic, organic layer, and novel heat-resistant substrate separators [495]. The inorganic modified separators are particularly favored in LIBs for their superior safety at high temperatures, while novel heat-resistant separators, made from materials like aramid and polyacrylonitrile (PAN), are emerging as a promising avenue for future development [496]. These separators are typically produced using techniques such as coating, grafting, electrospinning, and casting. By integrating biomaterials into the electrolyte and separator, researchers aim to create ESSs that are not only more durable but also more sustainable. This development is a direct response to the shortcomings of conventional liquid electrolytes, paving the way for environmentally sound solutions [497].

### 6.3. Integrating LIBs with Emerging Technologies: Internet of Things (IoT), Wearables, and Grid Storage

The rapid advancement of commercial electronics results in widespread application across various industries, including healthcare, sensing, the Internet of Things (IoT), sports, textiles, and more. The continuous improvements in electronic technologies drive this growth, enabling innovations and enhanced functionalities in these diverse fields [498]. The rise of electrification has been propelled by the introduction of LIBs, marking a significant advancement in rechargeable battery technology. Initially used in small portable electronics, LIBs have evolved to power EVs and larger stationary ESSs, showcasing their increasing versatility and importance in various sectors embracing sustainable energy solutions [499]. Electrochemical energy storage (EES) systems are widely acknowledged among the most advanced and efficient energy storage technologies available today. Among these, rechargeable LIB systems have become famous and the preferred choice for energy storage across many industries. The pervasive use of LIB systems highlights their superior performance and reliability, effectively addressing the energy storage demands of modern technologies and devices [500]. Out of power batteries such as lead-acid, nickel-hydrogen, LIBs, and fuel cells, LIBs stand out as the most commonly used. Their popularity is attributed to their high energy density, minimal self-discharge rate, and extended lifespan. Batteries are crucial in various sectors, including automotive, aerospace, industrial equipment, and ESSs, significantly impacting our daily lives. The advancement of technologies like artificial intelligence and big data has further enhanced the overall performance of batteries, leading to continuous improvements in their capabilities and efficiency [501]. While the review by Islam et al. [502] does not explicitly concentrate on batteries, it is essential to highlight that batteries are often combined with supercapacitors to achieve superior performance in many applications. This synergy between batteries and supercapacitors significantly enhances overall system performance and efficiency. LIBs, through their technological advancements, are poised to revolutionize the photovoltaic (PV) sector and sustainable energy, enabling broader access to essential services. While the economics of photovoltaics may not currently support expensive battery development, the progress in lithium battery technology is expected to reshape the industry. This growth extends to sectors like electric cars, electric bicycles (especially in China), and consumer electronics, driving down costs and increasing the feasibility of refillable lithium batteries for shared storage in off-grid renewable systems [503,504]. This connection underscores the importance of enhancing safety features and reducing costs to drive the broader adoption of LIBs, particularly in the rapidly growing EV market [505]. Chen and Ma [506] have focused on wearable LIBs utilizing carbon nanotubes and graphene. Their review examines the advancements in integrating these materials into wearable battery technology. Energy harvesting alone is often inadequate for IoT systems that require high power outputs, making specific wiring or batteries necessary. The primary focus for energy storage technologies in IoT applications is on integration-based solutions such as fuel cells, electrolyzes, lithium batteries, and supercapacitors [505]. While carbon nanotubes have been extensively explored, graphene shows promise with its superior electrical conductivity and potential for fabricating fiber-shaped LIBs. The focus on using carbon nanomaterials extends to other wearable battery types, such as lithium-air, sulfur, zinc-air, and aluminum-air batteries. Each battery type offers unique advantages but requires further research to improve its energy storage capabilities and mechanical stability [507].

LIBs can be significantly enhanced through the adoption of recent advanced technologies like IoT, wearables, and grid storage, which hold considerable promise [34]:

- Integration with IoT: Incorporating LIBs with IoT has the potential to power devices for extended periods wirelessly and autonomously. IoT envisions a comprehensive network where every object worldwide is interconnected, creating a unified ecosystem of interconnected devices and systems. This integration bridges the physical and virtual worlds, enabling seamless connectivity anytime and anywhere [507]. The use of IoT offers numerous advantages, including remote monitoring, data collection, and communication between interconnected devices. This leads to enhanced efficiency, productivity, and automation across various domains, such as smart homes, agriculture, healthcare, and industrial IoT.
- Wearable Devices: LIBs are excellent for powering wearable devices because they are compact, lightweight, and possess high energy density. Their integration into wearables, like fitness trackers, smartwatches, and medical devices, allows for continuous health monitoring, fitness tracking, and personalized healthcare solutions. This enhances the convenience and utility of wearables, providing users with reliable and extended power for their daily activities and health management.
- Grid-scale ESSs: LIBs are integral to grid-scale ESSs, providing essential stability and optimization to the electricity grid. By storing surplus energy when demand is low and releasing it during peak periods, they help to balance the load and ensure a reliable and steady power supply. This capability is crucial for maintaining grid efficiency and resilience, aiding the seamless integration of renewable energy sources, and improving overall grid performance.

Bi and colleagues [508] suggest integrating artificial intelligence (AI) and IoT for recommendation-based scenarios with low accuracy and recall in the scope of intelligent libraries. However, managing the large-scale and diverse data generated by IoT devices raises privacy concerns, so robust security measures, such as encryption and access control, have been introduced as crucial approaches for protecting sensitive information. Federated learning-based AI algorithms have found their place in the literature to reduce data exposure risks. However, more studies related to the convergence of AI, IoT, and emerging technologies like Digital Twins and Metaverse have been suggested in the cases of LIB applications in brilliant libraries, offering new solutions to address existing challenges and enhance user experiences. Ongoing research in the rapidly evolving field of electrochemical energy storage focuses on exploring new electrode and electrolyte materials, innovative designs, and 3D configurations at the nano-micrometer level to enhance power and energy density. As this research progresses, it is anticipated that optimized soft batteries will be developed, facilitating their integration into flexible devices. This advancement is expected to establish a more mature and specialized field within energy storage, paving the way for enhanced application performance and versatility.

### 6.4. Sustainable Battery Manufacturing

New research indicates that advancements in technology and economies of scale will make LIB manufacturing more energy-efficient in the future, despite rising global demand. This improvement is expected to mitigate concerns about energy consumption and greenhouse gas emissions [509]. LIBs have raised several sustainability concerns, particularly when examined over long-term applications. Recent studies highlight a significant conflict between performance-oriented parameters, like power density and energy density, and the selection of materials that prioritize sustainability. This conflict arises because high-performing batteries often rely on materials that pose environmental and ethical issues regarding their sourcing, production, and disposal. As a result, finding a balance between achieving optimal performance and adhering to sustainability principles remains a crucial challenge in the development and use of LIBs [508]. According to sustainable energy-storage methods, the cathode electrodes, such as Li–S and Li–O_2_ chemistries, show outstanding potential. Still, the wide-scale use of these batteries has to be further evaluated. In addition, in Li–S and Li–O_2_ batteries, different amounts of Li materials still are required to be used in the case of different electrodes and electrolytes. In the literature, different active materials have been introduced to provide significant benefits in the way of designing new battery chemistries as well as battery electrode materials [214]. Recently, many scholars have proposed the industrial and semi-industrial production of sustainable metal elements as an alternative to anode chemistries. For example, considerable and low-cost metallic elements, including Na and K, divalent Ca, Mg, and Zn, and trivalent Al, have found their place in the recent sustainable energy-storage technologies [510,511]. In addition, the common elements of C, H, O, N, P, and S can also be utilized in these batteries, known as environmentally friendly ones [453,511,512]. These biomaterials reduce the weight of batteries, resulting in high gravimetric energy density. The tunable structure of biomaterials allows for the elimination of electrochemically inactive sections and functionalization with desired groups. Additionally, they offer various advantageous properties, including sufficient ionic/electronic conductivity, minimal volume change, and exceptionally high specific capacities. Addressing these concerns is crucial for ensuring the long-term viability of LIB technology.

### 6.5. Recycling Practices

The LIB industry has seen rapid growth since it began in the late 20th century. Disruptive growth in technologies often happen when a product becomes easy to mass-produce, making it accessible to a larger market. Recycling is generally considered a final step in which as many materials as possible are returned to a circular economy. However, even as LIB development and commercialization continue to advance, the recycling industry is not keeping up with this progress [513,514,515]. Many electronic wastes (E-waste) have been generated in the last decades from end-of-life spent electronic-based (E-based) systems. The spent LIBs, as important E-waste, are increasing rapidly due to the rising trend of their worldwide applications in portable electronics, EVs, and renewable energy storage, leading to environmental and safety concerns because of various pollutants in the structure of these batteries [516]. The International Energy Agency (IEA) highlights that EVs manufactured in 2019 contributed approximately 500,000 tons of LIB waste, with projections indicating that by 2040, this cumulative waste could escalate to as much as 8 million tons [517]. The growing importance of LIB recycling is driven by technological advancements, valuable components, and environmental concerns. Governments must prioritize regulations to promote responsible recycling practices among manufacturers and users. While the US and EU are following China’s lead in this area, challenges remain in recycling processes, regulations, and timely implementation to minimize environmental risks. Exploring second life applications for batteries offers further opportunities for material recovery and sustainable LIB management [518]. The metal resources of LIBs show to have many economic potentials because of scarcity, increasing demand, and price, so by the recovery of these metal resources, namely Li, Co, and so on, the recycling process of spent LIBs may be justified [516]. Additional recycling facilities are required to manage this significant influx of LIB waste [465]. The growing adoption of LIBs has led to increased research efforts to explore practical and environmentally friendly methods for recycling spent LIBs. To foster a circular economy in the LIB market and mitigate environmental impacts, it is crucial to adopt sustainable recycling technologies [36]. While some previous studies have reviewed the status of LIB recycling technologies, there are relatively few comprehensive reviews that bring together both the technical aspects of recycling methods and the broader industry insights [517]. The increasing production and short lifespan of LIBs are leading to a growing battery waste issue. In response, states like California and New York have introduced legislation to ban the disposal of rechargeable batteries, such as the Rechargeable Battery Recycling Act of 2006. However, the recycling infrastructure to manage these batteries remains underdeveloped. While some companies have made progress in recycling processes and collection efforts, a comprehensive system for recycling end-of-life LIBs is still lacking, and the costs associated with establishing such infrastructure have not been thoroughly analyzed [519]. In the battery recycling industry, costs are influenced by several factors, including the chemical composition of the battery, the recovery rates at recycling facilities, and the potential for reuse of the materials contained within the batteries. NMC batteries, which contain high-value cobalt, are typically more attractive to recycling plants and hold considerable financial value through the recycling process. On the other hand, LFP batteries, which are less economically valuable, generally incur processing fees at recycling facilities. These factors indicate that the chemical composition of a battery plays a crucial role in determining recycling costs, which could lead to the establishment of a sustainable battery recycling industry [518]. Given the challenges and gaps in understanding highlighted in the broader e-waste literature, it is evident that a more proactive strategy is needed to establish a comprehensive recycling infrastructure for LIBs.

The Lander et al. [520] techno-economic model shows that LIB recycling can be profitable (ranging from −21.43 to +21.91 USD/kWh) but is highly dependent on factors like transport, wages, pack design, and recycling method. In-country recycling is suggested to reduce costs. The T&E model estimates that battery demand in Europe, driven by EVs and ESS, will approach 970 GWh by the end of the current decade and nearly double to around 2 TWh by 2040. This rising demand necessitates effective management and recycling of these batteries when they reach the end of their life, as they will become an important source of raw materials. The volume of batteries available for recycling is projected to increase significantly after 2030, with approximately 170 GWh expected in 2035 and growing to 470 GWh by 2040 [521]. Battery design is being revolutionized by using environmentally friendly and biodegradable materials, contributing to a more circular economy through reduced impact and improved component recyclability. Deep eutectic solvents (DESs) are emerging as a bioinspired solution for enhancing energy storage and battery recycling. These environmentally friendly solvents, created from hydrogen bond donors and acceptors, are less toxic and more biodegradable than conventional alternatives. Researchers are investigating DESs for use in redox flow batteries and LIB recycling due to their broad electrochemical windows and good ionic conductivity. While DESs can have higher viscosities that impact reaction rates, their sustainable and economical nature makes them a promising avenue for advancements in battery technology [522]. Renewable biobased materials offer promising alternatives to fossil-based components in LIBs and LSBs, potentially allowing for the substitution of around 25% of a battery’s mass with sustainable resources like biobased electrolytes, graphite electrodes, separators, and binders. Key materials under consideration include lignocellulosic biomass such as cellulose and lignin, sodium alginate extracted from seaweed, and chitin or chitosan derived from crustacean shells. These options are advantageous because they reduce environmental pollution and can be repurposed after their life cycle. Nonetheless, several challenges need to be addressed before fully embracing the sustainability potential of these biobased materials. Issues such as limited long-term stability and performance, land use concerns, and the balance between biodegradability and stability are critical factors to evaluate [523]. Wang et al. [524] concentrate on hydrometallurgical techniques in their review of spent LIB recycling processes. Svard et al. [525] highlight that while DESs are promising for eco-friendly LIB recycling due to their selective metal dissolution, they currently struggle to compete with mineral acids industrially. The research emphasis should shift toward optimizing DES reuse and recovery strategies to enhance their application beyond the lab. Zhang et al. [526] examine a range of recycling technologies for LIBs, assessing their efficiency, economic feasibility, and environmental effects. They propose a sustainable recycling strategy for components of spent LIBs and explore the future challenges and trends in the recycling sector. Wu et al. [527] address growing concerns about LIB waste and the associated supply risks of raw materials. They focus on direct recovery methods that reuse degraded materials, which can lower chemical and energy costs. The review outlines key steps for regenerating electrode materials and examines how understanding degradation mechanisms can guide effective regeneration strategies, promoting sustainability. Solberg et al. [528] explore the use of electrodialysis to purify and recycle antisolvents in LIB recycling. They present a non-equilibrium electrochemical model to enhance the design and efficiency of ion exchange membranes for optimizing water and ethanol separation. Their findings indicate the potential for increasing ethanol concentration and highlight new applications for critical metal mixtures. Wang et al. [529] highlight the urgent need for improved recycling of LIBs due to their growing use in portable electronics and EVs, as current recycling rates are very low. They criticize existing methods for their inefficiency and high costs. The authors advocate for the development of direct recycling methods as a promising alternative. Their review offers insights into current practices, limitations, and guidance for future sustainable recycling systems. Likewise, He et al. [530] provide an overview of advancements in the recovery of valuable metals from spent LIBs, yet their review is confined to pyrometallurgical and hydrometallurgical methods. While more intricate than pyrometallurgy, the hydrometallurgical process offers superior benefits, including enhanced metal recovery rates, operation under mild conditions, and minimal environmental impact [531]. The main focus of scholars in the case of physical chemistry is on the use of pyrometallurgy, hydrometallurgy, and electrometallurgy methods for recycling spent LIBs. Still, green chemistry has recently taken its place. The first part of the recycling process is to extract essential metals in cathodes and anodes of LIBs. Still, this part is only the recycling process, and the pretreatment of spent LIBs to remove black mass powder and deal with the hazardous environmental substances should also be taken into consideration [532]. As stated by Wang et al. [533], there are many side substances in the LIBs, such as polyvinylidene fluoride (PVDF), that are very difficult to be separating because of their stable structure and C-F bonds. One of the critical approaches that should be further considered is the use of new heating methods, such as microwave heating, to enhance the performance of LIBs recycling [534]. However, the process of microwave heating in the case of recycling spent LIBs is in the first step of development, and further research should be considered. The various recycling processes employed can recover valuable materials, as outlined in Table 12.

However, it is essential to note that these processes have limitations. Continuous research and development efforts are underway to optimize these recycling methods further. The primary areas of focus for improving recycling processes include increasing yield and purity, reducing raw material and energy consumption, and minimizing waste [535]. In summary, comparison of three battery recycling methods reveals the following [515,536,537]: The hydrometallurgical method excels in achieving high metal recovery rates and product purity while maintaining relatively low energy consumption and minimizing gaseous waste. However, significant wastewater generation and lengthy processing times pose challenges, requiring focused efforts on wastewater management and process optimization.

The pyrometallurgical method, while offering simple operations and shorter processing time with flexible input material requirements, suffers from low recovery rates, high energy consumption, and substantial gaseous emissions requiring expensive treatment. Furthermore, it fails to recover valuable metals like lithium and manganese. Challenges center on reducing energy consumption and pollution and potentially integrating hydrometallurgical techniques to improve recovery.

The direct recycling method stands out for its short processing time, energy efficiency, and environmental friendliness, achieving high recovery rates. However, it demands advanced equipment and stringent operational standards, potentially leading to incomplete recovery. To identify the most effective process chain for LIB recycling, it is essential to examine variations in both the composition of input materials and the scale of operations. This requires a thorough evaluation of techno-economic aspects alongside performance-related indicators, considering critical factors such as operational scale, transportation distances, and cobalt content. By analyzing these elements, stakeholders can pinpoint optimal recycling strategies that are tailored to specific conditions and material characteristics. A summary of various battery recycling methods, including their processes, advantages, and applicable contexts, is presented in Table 13.

While both pyrometallurgical and hydrometallurgical techniques are well-established approaches for recycling LIBs, they do not significantly decrease life-cycle greenhouse gas emissions. In contrast, direct cathode recycling offers greater potential for reducing emissions and enhancing economic feasibility [540]. Although hydrometallurgy generally entails lower energy consumption and reduced facility costs compared to pyrometallurgy, it requires larger amounts of reagents and water. An LCA comparing these two methods indicated that pyrometallurgical recycling often results in higher environmental risks across multiple impact categories, such as global warming potential, carcinogenic and non-carcinogenic effects, ozone layer depletion, and the creation of photochemical ozone. Conversely, hydrometallurgical recycling is associated with greater risk of freshwater and terrestrial acidification [541]. The environmental shortcomings of pyrometallurgy are primarily linked to its high energy requirements for high-temperature processes and the inefficiency in lithium recovery, which frequently ends up in slag that is either disposed of in landfills or used in low-value applications like construction [542]. Environmental impacts of LIB recycling processes stem from several key factors. These include energy consumption (and associated greenhouse gas emissions), incineration-related toxic gas emissions and CO_2_ production, landfill disposal of processing residues, and the use of hazardous chemicals requiring extensive post-processing and remediation. Mitigation strategies should prioritize controlling emissions and implementing robust waste management practices. A further significant concern is the risk of environmentally damaging illegal recycling, particularly in developing countries, often due to weak regulatory frameworks and inappropriate technologies. This poses substantial health and environmental risks to vulnerable populations. Both pyrometallurgical and hydrometallurgical recycling methods necessitate pretreatment stages before further processing can occur. A summary of pretreatment processes in hydrometallurgical and pyrometallurgical methods is given in Table 14.

**Figure 15:**
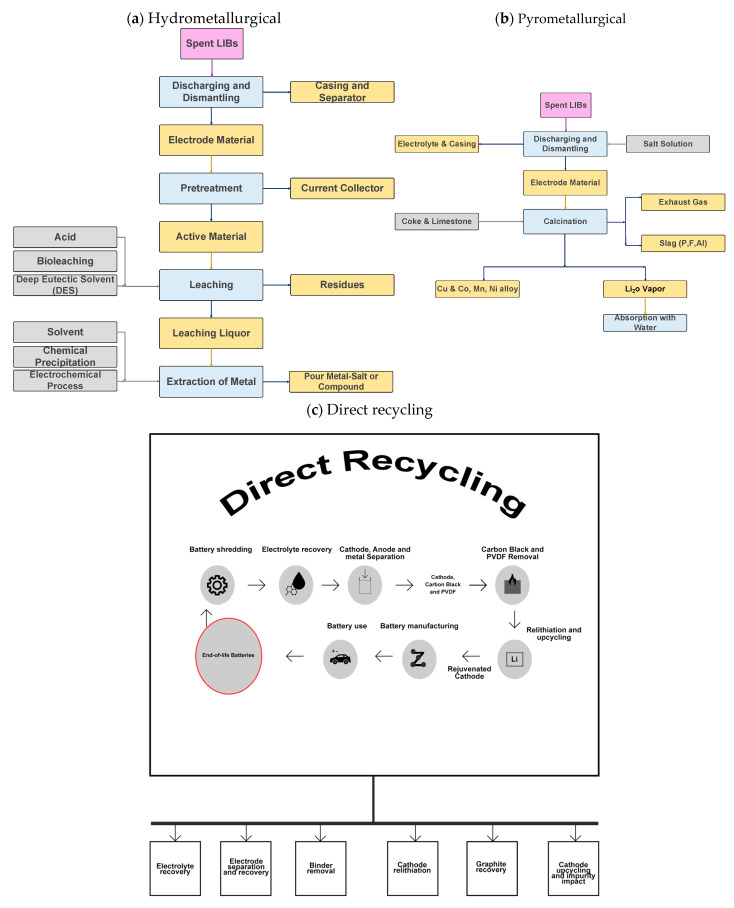
Diagrams of (**a**) Hydrometallurgical, (**b**) Pyrometallurgical, and (**c**) Direct Recycling: Hydrometallurgical: Uses aqueous solutions to leach out valuable metals from spent LIBs. Pyrometallurgical: Involves high-temperature processes to extract metals from spent LIBs. Direct: involves reusing the battery components or materials without extensive processing, for instance, reusing electrodes or other parts directly in new batteries [517].

A cost analysis of vehicle battery recycling methods suggests that hydrometallurgy becomes the most economically viable option at large-scale operations (above 20,000 metric tons capacity). Direct recycling and pyrometallurgy, in contrast, exhibit similar cost profiles for LIB recycling [467]. Figure 15 compares typical diagrams for hydrometallurgical, pyrometallurgical, and direct recycling procedures to recycle spent LIBs.

Key challenges for hydrometallurgy include reducing operational expenses, simplifying the types of input materials, and enhancing the quality of recovered products. Ultimately, the selection of an optimal recycling method necessitates a comprehensive evaluation of economic factors, environmental consequences, and the specific metals targeted for recovery. Table 15 presents factors influencing this decision. The practical application of recycling research hinges on industry adoption. This depends on several key factors, starting with demonstrable performance. The industry requires robust evidence that recycled materials meet or exceed the performance of virgin materials. Reliable supply chains are equally vital, ensuring consistent availability of recycled materials in sufficient quantity and quality. This necessitates developing robust logistics for spent batteries and scrap, potentially through partnerships between manufacturers and recyclers. Furthermore, the industry prefers processes aligned with existing practices, using common reagents and equipment, due to perceived risks associated with novel techniques. Historically, reluctance to use recycled materials, especially in high-performance applications, presents a challenge, requiring consistent adherence to stringent performance standards and a compelling cost advantage over virgin materials. While regulations could mandate recycling or recycled content use, a more sustainable approach focuses on economic incentives. Making recycling financially attractive reduces the need for regulatory intervention and fosters a market-driven transition towards a circular economy for battery materials. This involves demonstrating not only performance parity but also cost-effectiveness, thereby overcoming historical resistance and encouraging widespread adoption of recycled materials in even the most demanding applications. Table 16 summarizes the recent advances related to the use of different methods to recover wasted LIBs.

## 7. Future Considerations

- LIBs have revolutionized the portable electronics industry and are increasingly being adopted for EVs and grid storage. However, to fully appreciate future potential of LIBS, it is essential to compare them with other battery technologies. Table 17 compares LIBs with other battery technologies.
- Initially, the primary focus was on increasing the capacity of LIBs to provide longer usage times. However, there is now a growing demand for faster charging processes, as consumers desire greater portability and convenience by minimizing the time their devices spent connected to power sources. High-capacity LIBs require anode and cathode materials that effectively host lithium ions while maintaining a lightweight structure. Metallic lithium is impractical due to safety concerns, leading to the use of intercalation materials. The common cathode material, LiCoO_2_, has high theoretical capacity but can only utilize half of it safely. Additionally, the demand for lighter elements limits material choices and reduces the available space for Li-ion hosting [552]. Therefore, considerable research on the thermal behavior of LIBs during high or very high C-rate charge–discharge operations is essential.
- The future of biomaterial-based batteries hinges on developing stable, high-capacity cathodes and anodes, with a focus on bio-inspired materials like quinones, flavins, polydopamine, and amino acids. Enhancements in electronic conductivity, mechanical stability, and the use of multivalent ions in anodes present promising avenues for research. Additionally, biodegradable separators made from lignin and cellulose must achieve adequate ionic conductivity and strength, while bio-derived electrolytes like deep eutectic solvents show potential but need improvements in ionic mobility and stability. A major industrial challenge remains scaling production methods, necessitating cost-effective techniques such as 3D printing and green synthesis to maintain the performance and quality of materials in large quantities. Addressing these areas is key to realizing the potential of biomaterial-based ESSs.
- A significant challenge in LIB fabrication is developing high-capacity, long-cycling stable anode and cathode materials for greener energy storage [553,554]. A promising alternative to graphite anodes is biomass-derived carbon, which presents environmental benefits while addressing supply chain risks. Emphasizing the use of green binders and recycling can further minimize the carbon footprint of battery manufacturing, aligning with circular economy principles and promoting decarbonization in transportation. Initially, the primary focus was on increasing the capacity of LIBs to provide longer usage times. However, there is now a growing demand for faster charging processes, as consumers desire greater portability and convenience by minimizing the time their devices spent connected to power sources. High-capacity LIBs require anode and cathode materials that effectively host lithium ions while maintaining a lightweight structure. Metallic lithium is impractical due to safety concerns, leading to the use of intercalation materials. The common cathode material, LiCoO_2_, has a high theoretical capacity but can only utilize half of it safely. Additionally, the demand for lighter elements limits material choices and reduces the available space for Li-ion hosting [7].
- Future research should continue to optimize silicon-based anode materials to overcome current challenges, such as volume expansion and compatibility with graphite. Developing scalable manufacturing processes for these advanced materials is critical. Furthermore, the incorporation of nanomaterials in anode design appears to be essential, as they enable significantly enhanced intercalation and deintercalation rates compared to traditional anode materials. In addition, nanocarbon and nanostructured oxide materials provide advantageous properties such as increased surface area, which can further improve anode performance and efficiency.
- The future of electrolyte materials in LIBs hinges on addressing current limitations while enhancing overall performance. Ongoing research is focused on developing advanced solid-state and gel-based electrolytes that could improve ionic conductivity and mechanical strength, addressing issues of safety and battery longevity. Additionally, the integration of nanomaterials is poised to play a critical role in enhancing conductivity and stability, ultimately leading to safer, more efficient LIBs [20]. As innovations continue to emerge, the goal is to create electrolytes that not only optimize performance but also ensure higher safety standards to meet the demands of evolving energy storage applications, particularly in electrified transportation. These advancements are essential for unlocking the full potential of lithium-ion technology in the quest for sustainable energy solutions.
- Continued advancements in solid electrolytes are essential to realize their full potential in all solid-state batteries. Research should address ion conductivity and interface stability issues to enhance overall battery performance.
- Moving forward, research should aim to explore innovative cathode materials with improved energy densities, such as lithium-rich layered oxides and transition metal oxides. The impact of high-nickel materials must be understood, particularly regarding safety and thermal stability. Advances in composite cathode materials could combine the benefits of low-cost and abundant metals with high-capacity performance. Investigating the impacts of dopants and the use of nanostructured materials might yield significant gains in cycle life and capacity retention.
- Enhancing sustainability through effective recycling processes remains a key challenge in LIB technology. Research must focus on developing circular economy strategies that not only recover valuable metals but also design batteries for easier disassembly and recycling. Innovative recycling methods, such as direct recycling techniques that reuse battery components without extensive re-processing, should be examined for efficiency and scalability. Novel processes that leverage biotechnological methods for metal recovery and environmentally friendly solvents could significantly minimize the ecological footprint of battery production and disposal.
- Solid-state batteries have gained traction as a promising alternative to conventional LIBs, primarily due to their enhanced safety profiles and potential for higher energy densities. Researchers are actively investigating various solid electrolyte materials, including sulfides, oxides, and polymer-based electrolytes, to overcome the challenges associated with ionic conductivity and interfacial stability. Future research could focus on developing scalable production methods for solid-state technologies, optimized interfaces for solid electrolytes, and the integration of metallic lithium anodes, which could potentially double the energy density compared to traditional LIBs.
- As lithium resources become constrained [555], sodium-ion batteries present a viable alternative due to the abundance and low cost of sodium. Studies could explore novel oxides and polyanionic materials that could replace conventional LIB cathodes while achieving comparable energy densities. Additionally, understanding the sodium-ion diffusion and electrochemical mechanisms is crucial to enhancing LIB cycling performance and stability.
- Lithium sulfur batteries present a highly attractive energy-dense alternative, achieving specific energy densities exceeding 500 Wh/kg. However, challenges such as polysulfide dissolution and slow kinetics pose significant hurdles. Future research should focus on advanced cathode designs, including the use of conductive carbon nanocomposites to trap polysulfides and enhance their electrical conductivity. Novel electrolyte formulations that stabilize lithium polysulfides and materials for robust interface protection are also essential avenues. Meanwhile, lithium-air batteries, which can theoretically achieve even higher energy densities by using oxygen from the air, require breakthroughs in air cathode design, electrolyte stability, and management of side reactions. Research efforts should concentrate on improving efficiency and operational lifespan while tackling current challenges.
- The future of LIBs will increasingly involve smart battery management systems that employ AI and IoT technologies to optimize performance. Research could investigate the development of predictive algorithms that enhance battery life, provide real-time monitoring and diagnostics, and improve charge–discharge cycles. Integrating BMS with renewable energy sources and smart grids can facilitate more efficient energy usage across various applications, from EVs to large-scale energy storage systems.
- Future studies should employ advanced characterization techniques, such as operando and in situ methods, to gain deeper insights into the electrochemical processes occurring within LIBs. Understanding degradation mechanisms at the atomic level can lead to innovations in materials and designs that enhance battery lifespan and performance. Research could also explore how different testing conditions and preprocessing steps affect battery behavior under real-world scenarios.

## 8. Conclusions

The comprehensive analysis of LIB technology presented in this document underscores the pivotal role of continuous research and development in advancing energy storage solutions. The intricate balance of optimizing electrode materials, electrolytes, and separators is crucial for enhancing the performance and safety of LIBs. The document highlights significant strides in recycling technologies, essential to address the sustainability challenges posed by the growing use of LIBs. Efficient metal recovery methods and innovative recycling practices are critical to minimizing environmental impact and ensuring resource availability. Future research should focus on refining these recycling processes and developing new, eco-friendly materials to further reduce the ecological footprint of LIBs. The findings reinforce the necessity for a holistic approach that integrates technological innovation with sustainable practices, paving the way for more efficient, safe, and environmentally responsible energy storage solutions. This comprehensive review paper serves as a valuable resource, detailing not only the technical and scientific advancements but also the critical challenges faced by the industry.

## Figures and Tables

**Table 1:** KPIs for LIBs technology [31].

Specific Energy (Wh/kg)	Specific Power (W/kg)	Energy Density (kWh/m^3^)	Power Density (kW/m^3^)	Lifetime (Years)	Lifetime Cycle (Cycles)	Cell Voltage (V)	Efficiency (%)
80–250	200–2000	95–500	50–800	5–15	100–10,000	2.5–5	75–97

**Table 2:** Comparison of electrochemical reactions in a LIB during discharge and charge cycles.

Feature	Discharge	Charge
Anode (Negative Electrode)		
Material	Graphite (C_6_)	Graphite (C_6_)
Reaction	LiC_6_ → C_6_ + Li^+^ + e^−^ (Lithium is released from the graphite structure)	C_6_ + Li^+^ + e^−^ → LiC_6_ (Lithium is inserted into the graphite structure)
Process	Oxidation (Loss of electrons)	Reduction (Gain of electrons)
Cathode (Positive Electrode)		
Material	(LiCoO_2_)	(LiCoO_2_)
Reaction	LiCoO_2_ → Li_1−x_2__CoO + xLi^+^ + xe^−^ (Lithium is released from the oxide structure)	Li_1−x_2__CoO + xLi^+^ + xe^−^ → LiCoO_2_ (Lithium is inserted into the oxide structure)
Process	Reduction (Gain of electrons)	Oxidation (Loss of electrons)
Overall, Cell Reaction	LiC_6_ + LiCoO_2_ → C_6_ + Li_1−x_2__CoO	C_6_ + Li_1−x_2__CoO → LiC_6_ + LiCoO_2_
Li^+^ Ion Flow	Anode to Cathode (through electrolyte)	Cathode to Anode (through electrolyte)

**Table 3:** Comparison of three types of battery cells [129].

Cell Type	Advantages	Disadvantages
Cylindrical	- Dense packing capability - No additional system is needed to handle pressure changes during charging or discharging	- Complex design and battery management system - High weight-to-energy storage ratio
Prismatic	- Less complicated - High energy density - Ideal for heavy-duty EVs	- More losses in damage - Costlier than other types
Pouch	- Low weight	- The casing is not designed to protect the battery from heavy loads

**Table 4:** Comparison of different anode materials for LIBs. Material types, mechanisms, the anodes of rechargeable LIBs, advantages, and disadvantages for alloying, conversion, and intercalation-based anode materials.

Material Type	Mechanism	Advantages	Disadvantages
Alloying (e.g., Si, Sn, Ge, Sb)	Li-atom insertion and alloy formation.	- High theoretical capacity: Offers significantly higher energy density compared to graphite. - Abundant resources: Some materials, like Si, are readily available.	- Large volume changes: Severe expansion/contraction during cycling leads to rapid capacity fade, electrode pulverization, and short cycle life. - Low Initial Coulombic Efficiency (ICE): Significant irreversible capacity loss during the first few cycles. - Safety concerns: Potential for lithium dendrite formation and thermal runaway, especially at high Li content.
Conversion (e.g., Fe_2_O_3_, NiO)	Conversion reaction involving metal formation and Li_2_O.	- High theoretical capacity: Comparable to alloying materials, offering significant energy density improvement. - Abundant and low-cost materials: Many conversion materials are derived from earth-abundant and inexpensive metal oxides.	- Structural pulverization. - Electrical contact loss. - Large volume changes.
Intercalation (e.g., Graphite, Li_4_Ti_5_O_12_)	Reversible insertion of lithium ions into a host material’s structure without significant structural changes.	- Good cycle life: Demonstrates excellent long-term cycling stability with minimal capacity fade. - High-rate capability: Enables fast charging and discharging for high-power applications. - Mature technology: Graphite is widely used in commercial Li-ion batteries, with well-established manufacturing processes.	- Large volume changes. - Structural degradation. - SEI formation. - Safety concerns with graphite: At high charge rates, lithium plating can occur, posing safety risks.

**Table 8:** Factors affecting battery degradation from the perspective of EVs.

Level	Factors	Ref.
Design of the battery	- Materials - Electrodes - Cells - Battery management system	[265,266] [267,268] [269,270] [271,272]
Battery production	- Production stage issues.	[273,274,275,276,277]
Battery working condition	- Temperature - State of Charge (SOC) - Current - Aging	[278,279] [280,281,282] [283,284] [285,286]

**Table 10:** Applications of EIS for LIBs.

Application	Ref.
Characterization of Rb in EIS spectra	[312,313,314,315]
Characterization of RSEI in EIS spectra	[316,317]
Characterization of Rct in EIS spectra	[318]
Characterization of Warburg in EIS spectra	[301,319]

**Table 11:** Mitigation strategies to enhance the safety of LIBs.

Mitigation Strategy	Classification of the Leading Mitigation Strategies	Ref.
Innate safety strategies	- Anode Alteration (Protection) - Cathode Alteration - Electrolyte Alteration - Separators - Battery Management Systems	[400,401] [402,403] [404,405] [406,407] [408,409,410,411]
Safety devices	- Protection Vents - Positive Thermal Coefficient Device - Other Circuit Cut-off Devices	[412] [413] [414]
Thermal management	- Air Cooling of LIBs - Liquid Cooling of LIBs - Phase Change Material (PCM) Cooling of LIBs	[415,416] [417,418] [419,420]
Mechanical mitigation Strategies	- Battery Casing	[421]
Fire Control	- Fire Diagnosis Device - Fire Extinguishing	[422] [423,424]

**Table 12:** Materials recoverable through various recycling technologies [31].

Hydrometallurgical	Pyrometallurgical	Direct
- Copper - Steel - Aluminum - Graphite - Plastics - Lithium carbonate - Co^2+^ in output - Ni^2+^ in output - Mn^2+^ in output - Electrolyte solvents - Electrolyte salts	- Copper compounds - Iron compounds - Co^2+^ in output - Ni^2+^ in output - Lithium compounds - Aggregate (from slag)	- Copper - Steel - Aluminum - Graphite - Plastics - Electrolyte solvents - Electrolyte salts

**Table 13:** Overview of battery recycling methods [527,538,539].

Recycling Method	Process	Advantages	Context
Pyrometallurgical Recycling	High-temperature smelting of batteries	- Effective recovery of lead and nickel - Can handle various battery types	Ideal in regions with established high-temperature industrial infrastructure
Hydrometallurgical Recycling	Aqueous chemistry to leach metals	- High-purity selective recovery - Lower energy consumption compared to pyrometallurgy	Appropriate in areas with strict emissions regulations
Direct Recycling	Retrieval of intact active materials for reuse	- Preserves battery performance - Reduces dependence on virgin materials	Best for high-tech environments emphasizing sustainability and efficiency

**Table 14:** Pretreatment processes in battery recycling: A summary [543,544].

Process Stage	Description	Methods	Advantages	Disadvantages
Battery Sorting	Separates batteries based on type, health, size, and cathode material.	Manual sorting, machine-assisted sorting (robotic arms, automated assembly lines), X-ray scanning.	Improves efficiency of metal recovery, ensures purity of final product, facilitates downstream processes.	Manual sorting is labor-intensive and slow; automated methods can be costly to implement; electrolyte leakage and electrical risks pose safety concerns.
Battery Discharging	Safely removes residual electricity from batteries to prevent accidents during dismantling.	Liquid medium discharge (NaCl, KCl, sulfate solutions), solid medium discharge (graphite powder), pyrolysis/incineration, ultrasonic oscillation, inert gases.	Prevents leakage accidents, ensures safety.	Liquid media can be corrosive and expensive; solid media may pose thermal risks; discharge time can be long. Balancing cost-effectiveness and discharge efficiency is crucial.
Battery Disassembly	Separates battery components (Al casing, Cu foil, separator, current collector, active material) for reuse or further processing.	Manual disassembly, mechanically assisted disassembly (electronic clamps, robotic arms), collaborative robots.	Enables reuse of valuable components, prepares materials for downstream processing.	Manual disassembly is labor-intensive and slow; automated methods can be costly.
Battery Crushing and Flotation	Reduces batteries to small particles for easier metal extraction. Separates materials based on magnetic properties and particle size.	Dry crushing (direct shredding, cryogenic/inert atmosphere), wet crushing (with water and control reagents), magnetic flotation, sieving.	Increases surface area for metal extraction, facilitates separation of materials.	Dry crushing can lead to overheating, explosions, and dust generation; wet crushing requires handling of potentially hazardous solutions; particle size distribution can be inhomogeneous, requiring further separation.

**Table 15:** Key factors influencing the choice of battery recycling methods.

Factor	Influence on Choice
Economic Viability	Determines method suitability based on regional labor and energy costs.
Regulatory Environment	Stricter regulations can favor hydrometallurgy or direct recycling methods.
Material Composition	Influences the choice of method based on the specific types of batteries being recycled.
Technological Advances	Innovations may shift preferences toward more efficient or eco-friendly recycling options.

**Table 16:** Recent advances in Methods for recycling LIBs.

Method	Advantages	Disadvantages	Future Directions	Ref.
Hydrometallurgy, Pyrometallurgy, Direct Recycling	- Efficient metal recovery - High Ni, Co, Mn recovery with hydrometallurgy (>99%) - Handling mixed waste streams for market reintroduction - Efficient metal recovery with pyrometallurgy - Lower environmental footprint with direct recycling	- Battery sorting is crucial for hydrometallurgy and direct recycling - Safety considerations - Mechanical handling challenges in hydrometallurgy - Energy-intensive and emission-related issues in pyrometallurgy	- Scale-up evaluation - Solution metallurgy - Enhanced traceability for direct recycling - Metal separation pre- and post-recycling	[49,545,546]
Pyrometallurgical Recycling	- A robust method - Enabling scalability and sustainability	- Fluorine emission control and graphite, lithium, and manganese management issues - Inefficient lithium recovery	- Improved methods for pyrometallurgical fluorine emission management	[49,547]
Catalyst Synthesis	- Near 100% NOx conversion with high catalytic activity - Resource-efficient cathode material repurposing	- Synthesis process complexity: Morphology modulation challenges - Cost considerations: Material scalability and availability issues - Limited applicability beyond NOx conversion	- Industrial scale-up and cost optimization - Eco-friendly solvents and alternative waste sources usage	[548]
Mechanochemical Activation	- Accelerated leaching process: Reduced time, temperature, and acidic solvent usage - Improved S-LCO reaction activity: Refined particle size and enhanced surface energy - Excellent cycling performance and rate capability of regenerated LiCoO_2_ cells	- Scaling up MCA process complexity for industrial use - Further optimization is needed for broader applicability beyond LiCoO_2_ recycling	- Scalability and industrial viability of MCA process exploration. - Investigating co-grinding agent applicability to other battery chemistries - Optimization of MCA parameters for improved efficiency and environmental impact reduction	[549]
Graphite recovery and etching for lithium-sulfur cathode creation	- Utilization of spent graphite for resource conservation and waste reduction - Cathode with high initial capacity and stable performance across cycles - Cost-effective approach for designing sulfur-carbon composite cathodes	- Process complexity and environmental concerns with potassium hydroxide - Further optimization is needed for scalability due to limited characterization	- Optimization of recovery and etching processes for industrial scalability - Exploration of alternative, environmentally friendly etching methods. - Enhancement characterization for improved understanding and optimization - Integration of cathodes into battery systems for practical validation and improvement	[550]
Novel large-scale battery recycling: Pyro-hydrometallurgical approach, selective lithium leaching	- Efficient recycling process with high rates for lithium (94.47%) and transition metals (above 99% for Ni, Co, Mn) - Regenerated LiNi_0.6_Co_0.2_Mn_0.2_O_2_ shows comparable electrochemical properties to commercial counterparts - Economically viable with significant economic benefits (USD 20,667 per ton of recycled spent ternary cathode powder)	- Scaling up complexities for industrial application - Environmental concerns with high-concentration transition metal leachate usage	- Experimental condition optimization for efficiency and scalability enhancement - Exploration of alternative environmentally friendly leaching agents - Investigation into additional downstream applications for recycled materials beyond electrochemical properties - Integration of proposed recycling strategy into industrial settings for widespread implementation	[528]
Pyrometallurgical recycling routes	- Examination of pyrometallurgical recycling routes and battery compositions	- Profitability challenges and scalability limitations for low-cobalt/low-nickel batteries and related recycling processes	- Innovative tech/process optimizations for profitability challenges - Sustainable, cost-effective recycling for increasing electromobility demand	[551]

**Table 17:** LIBs in comparison with other battery technologies.

Feature	LIBs	Nickel-Cadmium Batteries	Nickel-Metal Hydride Batteries	Lead-Acid Batteries	Vanadium Redox Flow Batteries
Energy Density	High	Moderate	Moderate	Low	Low
Power Density	High	Moderate	Moderate	Moderate	Low
Cycle Life	Good	Good	Good	Moderate	Very Good
Cost	Moderate	Moderate	Moderate	Low	High
Safety	Good (with proper thermal management)	Good	Good	Good	Very Good
Memory Effect	None	Yes	None	None	None
Temperature Effect	Moderate	Moderate	Moderate	High	Low

## Data Availability

The original contributions presented in the study are included in the article, further inquiries can be directed to the corresponding author.
